# GDMT Intensity at Hospital Discharge and Associated Clinical Outcomes in Heart Failure: A Systematic Review and Network Meta-Analysis

**DOI:** 10.3390/jcm15135112

**Published:** 2026-07-01

**Authors:** Sergio Alejandro Gómez-Ochoa, Lyda Z. Rojas, Carlos A. Corona-Arias, Lizeth N. Quiroga-Pico, Laura V. Arciniegas-Landínez, Angie Yarlady Serrano-García, Angie C. Mendoza-Quiñonez, Katherin A. Gamboa, Alexandra Contreras, Juliana Alexandra Hernández Vargas, Silvia Juliana Trujillo-Cáceres, Luisa Aguilera, Luis E. Echeverría

**Affiliations:** 1 Heart Failure and Heart Transplant Clinic, Fundación Cardiovascular de Colombia, Floridablanca 681004, Colombia; lizethquiroga@fcv.org (L.N.Q.-P.); angieserranogarcia@fcv.org (A.Y.S.-G.); 2 Department of General Internal Medicine and Psychosomatics, Heidelberg University Hospital, 69120 Heidelberg, Germany; 3 Nursing Research and Knowledge Development Group (GIDCEN-FCV), Research Center, Fundación Cardiovascular de Colombia, Hospital Internacional de Colombia, Piedecuesta 681017, Colombia; lydarojas@fcv.org (L.Z.R.); angiecristina799@gmail.com (A.C.M.-Q.); julianahernandezv@gmail.com (J.A.H.V.); silviaj.trujilloc@gmail.com (S.J.T.-C.); 4 Faculty of Health Sciences, Universidad Industrial de Santander, Bucaramanga 680002, Colombia; carloscoronarias@gmail.com (C.A.C.-A.); lalavale0608@gmail.com (L.V.A.-L.); 5 Faculty of Medicine, Universidad de Boyacá, Tunja 150003, Colombia; kathemed.03@gmail.com; 6 Hospital General Regional 1, Instituto Mexicano del Seguro Social, Querétaro 76000, Mexico; alexacl9908@gmail.com; 7 Department of Global Public Health and Bioethics, Julius Center for Health Sciences and Primary Care, University Medical Center Utrecht, 3584 CX Utrecht, The Netherlands; 8 Heart Failure and Transplant Clinic, Instituto Cardiovascular de Mínima Invasión, Guadalajara 45116, Mexico; luisaaguilera1208@gmail.com

**Keywords:** guideline-directed medical therapy, heart failure, network meta-analysis, hospital discharge, mortality, SGLT2 inhibitors

## Abstract

**Background/Objectives**: Contemporary heart failure (HF) guidelines recommend early initiation of four foundational drug classes in HFrEF. However, real-world prescription rates of guideline-directed medical therapy (GDMT) at discharge remain low, and comparative data in this setting is limited. We aimed to explore the association between GDMT intensity prescribed at or before hospital discharge and clinical outcomes. **Methods**: MEDLINE and EMBASE were searched through March 2026 (PROSPERO CRD420261352137). A frequentist random-effects network meta-analysis grouped regimens into four intensity nodes (single/none, double, triple, quadruple), with the primary analysis restricted to adjusted hazard ratios. The primary outcome was the composite of all-cause mortality (ACM) and HF hospitalization (HFH). Secondary outcomes were HFH alone, ACM, and cardiovascular mortality. Confidence was rated with CINeMA. Estimates are reported as associations, not treatment effects. **Results**: Twenty-seven studies (26 observational, 1 RCT; 73,174 patients) were included. Among SGLT2i-era cohorts, quadruple therapy was suboptimally prescribed (pooled 34%, range 11.6–55.2%). For the primary composite endpoint, more complete regimens were associated with progressively lower event rates versus single or no therapy (double: hazard ratio 0.76, 95% CI 0.68–0.84; triple: 0.72, 0.64–0.81; quadruple: 0.52, 0.36–0.75; τ^2^ = 0). A consistent ordinal gradient was seen across outcomes, with quadruple therapy ranking first numerically for every outcome in which it was estimable. The direction and ordering were preserved in a sensitivity analysis additionally incorporating risk ratios, on stratification by follow-up duration, and in an alternative network anchored to incomplete therapy. Because most evidence was observational, the magnitude of these associations should not be interpreted as a causal treatment effect and likely reflects residual confounding and selection bias. **Conclusions**: Discharge GDMT remains an important opportunity to improve outcomes in patients with HF. Adequately powered randomized trials are required to establish the incremental benefit of this approach and to close the gap between evidence and practice.

## 1. Introduction

Heart failure (HF) remains a leading cause of morbidity, mortality, and healthcare expenditure worldwide [1]. Landmark randomized controlled trials (RCTs) over the past three decades have established the mortality and morbidity benefits of four foundational pharmacologic classes, beta-blockers, angiotensin-converting enzyme inhibitors (ACEi)/angiotensin receptor blockers (ARBs)/angiotensin receptor-neprilysin inhibitors (ARNi), mineralocorticoid receptor antagonists (MRAs), and sodium–glucose cotransporter 2 inhibitors (SGLT2i), in patients with heart failure with reduced ejection fraction (HFrEF) [2]. Acting through complementary neurohormonal pathways, these agents deliver independent and additive reductions in all-cause mortality (ACM) and heart failure hospitalization (HFH), underpinning the rationale for combined foundational therapy [2,3]. These four pillars of guideline-directed medical therapy (GDMT) are now recommended as Class I therapy for HFrEF in international guidelines, and analyses including trials in the outpatient setting have shown that the quadruple combination provides the greatest survival benefit, with an estimated up to eight additional life-years compared with no treatment [4,5].

Despite this robust evidence, real-world registry data consistently demonstrate that comprehensive GDMT is suboptimally prescribed to eligible patients at hospital discharge [6,7]. This implementation gap is multifactorial, driven mainly by clinical inertia, but also including concerns about hemodynamic tolerability, fragmented care transitions, and the complexity of initiating multiple medications during an acute hospitalization [8,9]. The STRONG-HF (Safety, Tolerability, and Efficacy of Rapid Optimization, Helped by NT-proBNP Testing, of Heart Failure Therapies) trial provided the first randomized evidence that rapid, intensive up-titration of GDMT before discharge was safe and reduced the composite of HF rehospitalization and ACM at 180 days [10]. However, STRONG-HF did not include SGLT2i in its protocolized regimen and enrolled patients across the ejection fraction spectrum, leaving uncertainty about the incremental benefit of true quadruple GDMT at discharge [10].

A growing body of observational evidence has emerged comparing clinical outcomes across different GDMT intensity levels, from single-agent or no therapy through double, triple, and quadruple therapy, in patients discharged after an HFH. These studies have consistently reported dose–response associations favoring more complete GDMT, but they vary substantially in design, populations, follow-up duration, and definitions of GDMT intensity. To address these gaps, we performed a systematic review and network meta-analysis (NMA) to synthesize and explore the associations between GDMT intensity at hospital discharge and subsequent clinical outcomes.

## 2. Materials and Methods

This study was registered in PROSPERO (CRD420261352137) and performed and reported in accordance with the Preferred Reporting Items for Systematic Reviews and Meta-Analyses (PRISMA) 2020 guidelines and the PRISMA extension statement for network meta-analyses (Supplemental Table S1) [11,12]. Institutional Review Board approval was not required, as all data were previously published.

**Search Strategy and Study Selection.** We performed systematic searches of the PubMed/MEDLINE and EMBASE Elsevier databases from inception through March 2026 for studies assessing clinical outcomes after HFH, stratified by the intensity of GDMT prescribed at or before discharge. The search strategy combined four conceptual blocks: (1) HF and related terms; (2) hospitalization and discharge-related terms; (3) pharmacotherapy terms covering GDMT pillars and combination therapy; and (4) timing, optimization, and initiation strategy terms. Complete search strings are provided in Supplemental Texts S1 and S2. No language or date restrictions were applied. Twelve investigators independently screened titles and abstracts in pairs, with conflicts resolved by consensus with a third reviewer. Study screening was performed using the Rayyan^®^ (Rayyan Systems Inc., Cambridge, MA, USA) [13].

Because study-level regimen reporting was heterogeneous, treatment nodes were harmonized pragmatically. The single/none reference node comprised patients prescribed no foundational class or a single class; because the included studies seldom reported a pure untreated arm separately from a single-agent arm, this category was harmonized as the least-intensive group reported by each study, and a network separating untreated from single-agent patients was not estimable, as too few studies reported the zero-agent and single-agent strata separately. The network should therefore be interpreted as comparing GDMT intensity categories rather than identical pharmacologic interventions across studies.

**Data Extraction and Treatment Classification.** Study-level data were extracted by four investigators and included: study design, country, sample size, patient demographics, left ventricular ejection fraction (LVEF) criteria, GDMT components, follow-up duration, and effect estimates with 95% confidence intervals (CIs). When studies reported multiple effect estimates for the same comparison, we prespecified the extraction of the most fully adjusted model available, prioritizing estimates derived from multivariable Cox proportional-hazards regression or propensity-score methods over unadjusted estimates. Crude estimates were extracted only when adjusted measures were not reported, and this occurred almost exclusively for risk ratios derived from published 2 × 2 event tables [14,15,16,17].

**Outcomes.** The primary outcome was the composite of ACM and HFH. Secondary outcomes included ACM, HFH, and cardiovascular (CV) mortality.

**Risk of Bias Assessment.** Observational studies were assessed at the study level using the Newcastle-Ottawa Scale (NOS), while the single included RCT (STRONG-HF) was assessed using the Cochrane Risk of Bias 2 (RoB 2) tool [18,19]. Confidence in network estimates, on the other hand, was assessed using the CINeMA framework across six domains: within-study bias, reporting bias, indirectness, imprecision, heterogeneity, and incoherence [20]. For CINeMA, risk of bias (RoB) assessments of observational studies were set to high within-study bias because treatment allocation was nonrandomized and highly susceptible to residual confounding by indication. Downgrading to moderate was allowed only if prespecified design safeguards were present, including exceptionally strong confounder control, clear exposure definition, robust outcome ascertainment, and minimal risk of selection bias. No study fulfilled these criteria sufficiently to warrant downgrading. These study-level judgments were then combined with the percentage contribution matrix to derive comparison-level assessments across all six CINeMA domains. Imprecision was evaluated against a prespecified equivalence range defined by clinically important risk ratio thresholds of 0.90 and 1.11. Six investigators independently assessed RoB, with disagreements resolved by consensus.

A frequentist random-effects NMA was performed using the netmeta package (version 3.3–1) in R version 4.5.1 (R Foundation for Statistical Computing, Vienna, Austria) [21], implementing the graph-theoretical approach [22]. Treatment effects were modeled on the log-ratio scale, with single/none therapy as the reference group. Because most included studies were nonrandomized, network estimates were interpreted as associations rather than causal treatment effects. Because hazard ratios (HRs) account for the time-to-event structure and censoring of the included cohorts and were predominantly derived from multivariable- or propensity-adjusted models, the primary network was restricted to adjusted HRs, and estimates are reported as hazard ratios. As a sensitivity analysis, we additionally fitted a combined network incorporating both HRs and risk ratios (RRs) on a common log-ratio scale (the “all-measures” network, reported as relative effects); because pooling HRs with RRs is valid only under low event rates and approximately proportional hazards, this network is presented as supportive rather than primary.

For multi-arm evidence structures, complete multi-arm studies were retained as true multi-arm designs within the network model, allowing the standard netmeta handling of within-study correlations [23]. When a study contributed an incomplete set of eligible multi-arm contrasts for a given outcome and time window, those contrasts were split into pseudo-two-arm entries for analysis. This approach preserved available information while allowing network estimation, but the resulting primary network estimates should be interpreted as average relative-effect estimates across heterogeneous study designs, regimens, and follow-up horizons.

**Transitivity and similarity across studies.** The validity of indirect comparisons in a NMA rests on the transitivity assumption, which states that studies contributing to different comparisons are similar in the distribution of effect modifiers. Because intensity nodes were harmonized pragmatically across heterogeneous designs, we evaluated transitivity both conceptually and empirically by compiling the distribution of potential effect modifiers (mean age, proportion of female participants, mean LVEF, geographic region, follow-up duration, publication era, and the prevalence of diabetes and chronic kidney disease, mean eGFR, and admission systolic blood pressure) across studies grouped by the most intensive regimen evaluated (the index node).

We also explored temporal heterogeneity by stratifying the primary 4-node ACM network by publication era (pre-2021 vs. ≥2021), as 2021 marked the widespread adoption of SGLT2i as a fourth pillar of HFrEF therapy. A formal network meta-regression on publication year was considered but not performed, because the quadruple-therapy node was informed exclusively by studies published from 2021 onward; publication year is therefore collinear with the availability of the most intensive node, precluding separation of an era effect from the treatment effect for that node. For clinical interpretation, HR-only network estimates for the composite of ACM and HFH were translated, consistent with GRADE guidance for binary outcome summary-of-findings tables [24], into illustrative 12-month absolute risks by applying each HR to an assumed baseline 1-year composite risk of 40% (treated risk = 1 − (1 − baseline)^HR^), with additional scenarios spanning 20% to 50%, to derive illustrative absolute risk reductions and numbers needed to treat for double and triple therapy. An absolute translation was not derived for quadruple therapy, whose composite estimate is informed by a single direct study. These estimates were not intended to represent causal risk reductions.

**Heterogeneity and inconsistency.** Heterogeneity was quantified using τ^2^ and Cochran’s Q statistic. Global inconsistency was assessed using design-based decomposition of the Q statistic; local inconsistency was explored using node-splitting (SIDE approach) [25], which compares direct and indirect evidence for each splittable comparison. Small-study effects and potential reporting bias were assessed visually using comparison-adjusted funnel plots with treatments ordered by therapeutic intensity (single/none → double → triple → quadruple), such that asymmetry to the left would indicate small-study effects favoring more intensive therapy. Given the limited number of studies per comparison (≤10 for most contrasts), formal regression-based asymmetry tests were not performed. Treatment hierarchies were assessed using P-scores [26]. Certainty of evidence was assessed using CINeMA [20]. All analyses used R version 4.5.1 (R Foundation for Statistical Computing, Vienna, Austria). Two-sided *p*-values < 0.05 were considered significant.

## 3. Results

**Figure 1:**
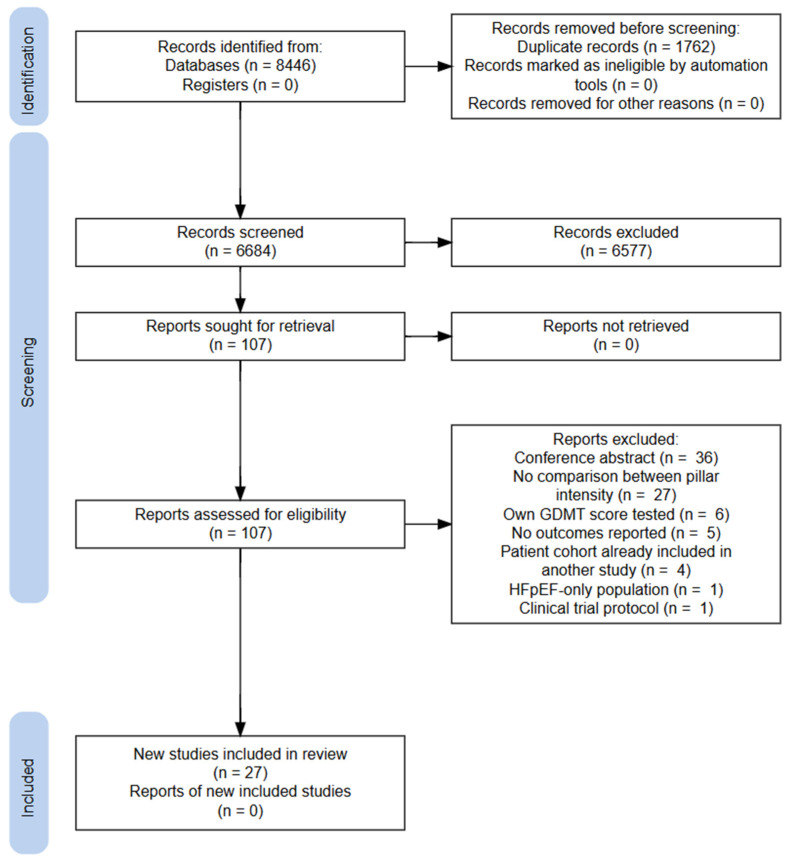
PRISMA Flow Diagram of Study Selection.

Twenty-seven studies met the inclusion criteria, comprising 26 observational cohort studies and one RCT (STRONG-HF), enrolling a total of 73,174 patients (Table 1 and Figure 1) [10]. Complete references of the included studies can be found in Supplemental Text S3. Studies were published between 2015 and 2026 across 14 countries, with the largest geographic representation from Asia (15 studies, 56%), followed by Europe and North America (five studies each), with single studies from South America and a multinational trial (STRONG-HF). The median sample size was 773 (interquartile range [IQR]: 479–1465), the mean age across studies was 69.5 years, 35.3% were female, and the mean LVEF was 29.7%. The median follow-up was 12 months (range: 1–43 months). Nineteen studies (70.4%) included exclusively patients with HFrEF (LVEF < 40%), while six (22.2%) included patients with LVEF < 50% (HFrEF and HFmEF populations), and two (7.4%) had no LVEF restriction (Table 1). The distribution of studies by geography, publication year, HF phenotype, and follow-up duration is summarized graphically in Supplemental Figure S1. Constituent drug-class composition and ARNI frequency by treatment node are summarized in Supplemental Table S2. The proportion of patients discharged on all four GDMT pillars was low and highly variable across contemporary cohorts published after SGLT2i approval for HF, ranging from 11.6% to 55.2% (median 43.8%). In a random-effects single-arm proportion meta-analysis using the Freeman–Tukey double-arcsine transformation, the pooled prevalence of quadruple therapy at discharge was 34.2% (95% CI, 21.3–48.5%) among these cohorts, with very high between-study heterogeneity (I^2^ = 99.2%; τ^2^ = 0.038; Q = 777.2, df = 6, *p* < 0.001).

Twenty of the 26 observational studies were rated high quality (NOS 7–9) and six moderate quality (NOS 5–6) (Supplemental Table S3). The favorable NOS profile should not be interpreted as high causal certainty, as the NOS does not fully capture biases relevant to causal treatment-effect estimation. Specifically, the NOS appraises the methodological conduct of a cohort study, whereas the CINeMA within-study-bias domain appraises the credibility of the nonrandomized treatment-effect estimate contributing to the network; a well-conducted cohort can therefore score highly on the NOS yet remain at high risk of confounding by indication, consistent with the treatment of confounding under ROBINS-I. Because GDMT intensity at discharge was not randomly assigned, observational comparisons remained susceptible to residual confounding, confounding by indication, and selection bias; in CINeMA, all observational studies were therefore classified as high within-study bias. STRONG-HF was judged to have some concerns on RoB 2 for the primary composite outcome, mainly related to the open-label design.

**Figure 2:**
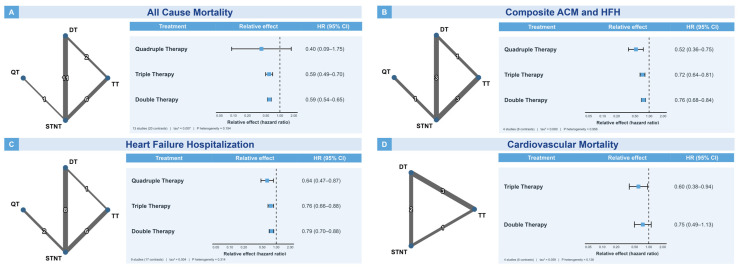
GDMT Intensity at Discharge and Clinical Outcomes after Acute Decompensated Heart Failure Hospitalization. Network (**left**) and Forest (**right**) plots showing the evidence structure and pooled network meta-analysis estimates for each of the four outcomes: all-cause mortality (**A**), composite of all-cause mortality and heart failure hospitalization (**B**), heart failure hospitalization (**C**), and cardiovascular mortality (**D**). Node size is proportional to the number of study arms, and edge width is proportional to the number of direct comparisons. All networks include four nodes: quadruple (QT), triple (TT), double (DT), and single/none (STNT) therapy. ACM = all-cause mortality; CV = cardiovascular; DT = double therapy; HFH = heart failure hospitalization; QT = quadruple therapy; STNT = single/none therapy; TT = triple therapy. CI = confidence interval; GDMT = guideline-directed medical therapy.

The primary (HR-only) analysis included 13 studies (20 contrasts) for ACM, four studies (eight contrasts) for the composite outcome, nine studies (17 contrasts) for HFH, and four studies (six contrasts) for CV mortality (Figure 2, Table 2 and Table S4). In the HR-only network the quadruple node was connected to single/none therapy for ACM, the composite, and HFH, but not for CV mortality, where it is reported only in the all-measures sensitivity network. All estimable networks were connected through at least one pathway.

**Figure 3:**
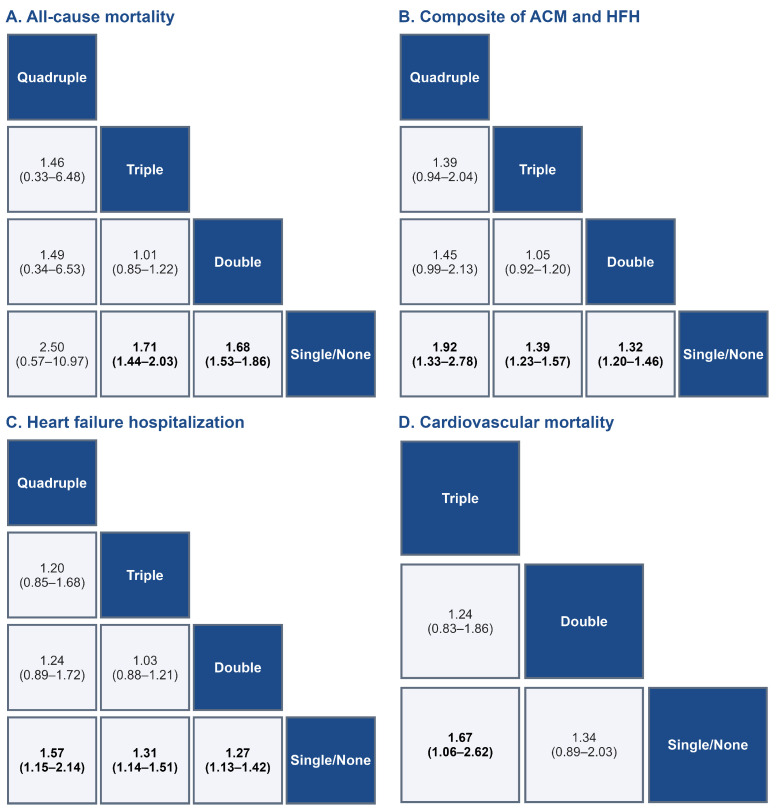
League Tables for All Primary Outcomes. Four-by-four league tables displaying all pairwise comparisons from the primary random-effects (adjusted HR) network meta-analysis for (**A**) all-cause mortality, (**B**) composite of ACM and HFH, (**C**) heart failure hospitalization, and (**D**) cardiovascular mortality. Values represent hazard ratios (95% CI) for the row treatment versus the column treatment. Statistically significant comparisons are highlighted. Because most evidence was nonrandomized, pairwise estimates should be interpreted as associations rather than causal effects. Abbreviations: ACM = all-cause mortality; HFH = heart failure hospitalization.

**Figure 4:**
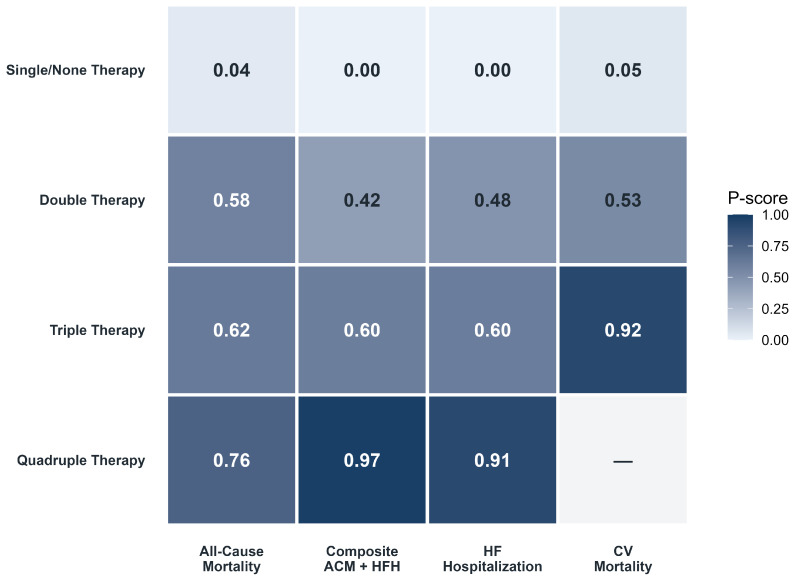
Treatment Hierarchy Across Clinical Outcomes. Heatmap showing P-scores from the primary random-effects network meta-analysis for quadruple, triple, double, and single/none GDMT at hospital discharge across the four primary outcomes: (i) all-cause mortality, (ii) composite of all-cause mortality and heart failure hospitalization, (iii) heart failure hospitalization, and (iv) cardiovascular mortality. Higher P-scores indicate a greater probability of ranking best within each outcome, whereas lower P-scores indicate a lower ranking probability. This figure summarizes the relative treatment hierarchy across outcomes and should be interpreted as a ranking metric rather than a measure of treatment effect magnitude or statistical significance. ACM = all-cause mortality; CV = cardiovascular; GDMT = guideline-directed medical therapy; HFH = heart failure hospitalization.

Compared with single/none therapy, the pooled hazard ratio was 0.52 (95% CI: 0.36–0.75) for quadruple therapy, 0.72 (0.64–0.81) for triple therapy, and 0.76 (0.68–0.84) for double therapy (Figure 2 and Figure 3). Table 3 presents an illustrative analysis of the absolute risk reduction and number needed to treat for medical therapy at discharge. Heterogeneity was not detected in this network (τ^2^ = 0; *p* for heterogeneity = 0.96), and quadruple therapy ranked highest (P-score = 0.98) (Figure 4). CINeMA confidence ratings were low for four comparisons and very low for two (quadruple vs. single/none, with only one study providing direct evidence, and quadruple vs. triple therapy, which relied entirely on indirect evidence and was downgraded for major imprecision) (Supplemental Tables S5 and S6).

In the primary HR-only network, progressively higher GDMT intensity categories were associated with lower ACM estimates versus single/none therapy, although the quadruple estimate was imprecise. The pooled HR was 0.40 (95% CI: 0.09–1.75) for quadruple therapy, 0.59 (0.49–0.70) for triple therapy, and 0.59 (0.54–0.66) for double therapy (Figure 2 and Figure 3). The quadruple-versus-single/none estimate was informed by a single direct study and its confidence interval crossed unity.

Heterogeneity was low (τ^2^ = 0.007; *p* for heterogeneity = 0.19), and treatment ranking favored quadruple therapy (P-score = 0.76), followed by triple therapy (0.62), double therapy (0.58), and single/none therapy (0.04) (Figure 4). In the primary HR-only network, confidence was low for the double-versus-single/none and triple-versus-single/none comparisons and very low for the remaining four; the quadruple-versus-single/none comparison was additionally downgraded for serious imprecision, as its single direct study yielded a wide interval crossing unity (0.40, 95% CI 0.09–1.75). Downgrading was otherwise driven by within-study bias (a major concern for all comparisons given the observational evidence base), suspected reporting bias, and indirectness arising from variation in population definitions and treatment constructs across studies (Supplemental Tables S5 and S6).

In the all-measures sensitivity analysis (combining HRs and RRs), the ordinal gradient persisted, but the quadruple-versus-single/none estimate was substantially more extreme (relative effect 0.13, 95% CI 0.06–0.28). This value was driven almost entirely by one small study reporting crude, unadjusted RRs (Huang 2025 [14], N = 343, 36-month follow-up); in the leave-one-study-out analysis, omitting this study alone returned the estimate to 0.40 (Supplemental Tables S7 and S8). The magnitude of the all-measures quadruple estimate should therefore not be interpreted as a treatment effect.

The pooled HR versus single/none was 0.64 (95% CI 0.47–0.87) for quadruple, 0.76 (0.66–0.88) for triple, and 0.79 (0.70–0.88) for double therapy (Figure 2 and Figure 3). Heterogeneity was low (τ^2^ = 0.004; *p* = 0.31), and the hierarchy again favoured quadruple therapy (P-score 0.92) (Figure 4). CINeMA confidence was low for the quadruple vs. single/none comparison and very low for the remaining five, reflecting heterogeneity (particularly for double vs. single/none, where the prediction interval extended substantially beyond the confidence interval) and imprecision in the indirect quadruple comparisons (Supplemental Tables S5 and S6).

In the primary HR-only network the quadruple node was not connected to single/none therapy for CV mortality (the only adjusted-HR quadruple datum derived from a comparison with incomplete therapy); the pooled HR versus single/none was 0.60 (95% CI 0.38–0.94) for triple and 0.75 (0.49–1.13) for double therapy, and triple therapy ranked highest among estimable nodes (P-score 0.92). In the all-measures sensitivity network, the quadruple-versus-single/none relative effect was 0.08 (0.02–0.40), but with very wide intervals and reliance on crude RRs (Figure 2 and Figure 3; Supplemental Table S7). Heterogeneity in the all-measures CV network was moderate (τ^2^ = 0.076; *p* = 0.069). Among the estimable comparisons, CINeMA confidence was low (triple vs. single/none) to very low (double vs. single/none and triple vs. double), with imprecision and heterogeneity the principal concerns; the three quadruple-therapy comparisons were not estimable in the HR-only network (Supplemental Tables S5 and S6).

Regarding transitivity assessment, studies informing the quadruple-therapy node enrolled younger patients (mean age 66 versus 69 and 75 years for the triple- and double-therapy nodes), were published exclusively from 2021 onward, and had shorter follow-up and higher mean eGFR, whereas sex and mean LVEF were comparable across nodes. These imbalances are consistent with evolving discharge practice in the SGLT2i era and represent potential sources of intransitivity; comparisons involving the quadruple node, which is informed solely by contemporary cohorts, should therefore be interpreted with particular caution (Supplemental Table S9).

Overall, the primary NMA suggested a consistent ordinal gradient across all four clinical outcomes, with progressively more complete GDMT associated with progressively lower relative event rates.

### 3.1. Time-Stratified Sensitivity Analyses

The direction of estimates was consistent across prespecified time windows. For ACM in the all-measures model at the intermediate 6–12-month horizon, relative effects versus single/none were 0.081 (quadruple), 0.495 (triple), and 0.539 (double); at long-term follow-up (>12 months), the gradient persisted (0.400, 0.565, 0.696), although the quadruple estimate was based on fewer studies and wider CIs. Composite and HFH sensitivity analyses preserved the ordinal pattern; some strata were informed by only 1–4 studies. CV mortality estimates remained directionally consistent but less precise (Supplemental Table S7).

Node-splitting revealed local inconsistency in one contrast of the all-measures ACM network: the double vs. quadruple comparison showed disagreement between direct (relative effect 8.33; 95% CI 3.23–21.5, derived from a single study, Huang 2025 [14]) and indirect (1.43; 95% CI 0.38–5.41; P for inconsistency = 0.034) evidence (Supplemental Figure S2). This contrast reflects the same crude RR study that drove the extreme all-measures quadruple estimate, and the inconsistency was attenuated in the primary HR-only network, in which no comparison was downgraded for incoherence (Supplemental Tables S5 and S6). No other ACM contrasts showed significant local inconsistency, and the composite outcome network showed no detectable heterogeneity or between-design inconsistency. The HFH network showed a statistically significant between-design component in the Q-decomposition (Q = 12.2; *p* = 0.016), which was attenuated when restricting to HR-only evidence (*p* = 0.469). The CV mortality network contained no splittable contrasts and was assessed at the global level only (Supplemental Table S11). Leave-one-study-out analyses for ACM showed that the direction and ordering of estimates were preserved when any single study was omitted, although the magnitude of the quadruple-versus-single/none estimate remained sensitive to individual studies (Supplemental Table S8).

### 3.2. Alternative Network Analysis

With incomplete therapy as the reference (excluding single/none), the gradient was broadly preserved. In the HR-only alternative network, the HR versus incomplete therapy for the composite endpoint was 0.50 (95% CI 0.34–0.73) for quadruple, 0.61 (0.46–0.82) for triple, and 0.63 (0.44–0.90) for double therapy; for ACM it was 0.63 (0.46–0.85) for triple and 0.74 (0.46–1.18) for double therapy, with the quadruple node not estimable against incomplete therapy in the HR-only network (the corresponding all-measures estimate was 0.14, 0.04–0.49). The alternative HFH and CV mortality networks showed greater heterogeneity and wider confidence intervals and are therefore regarded as supportive rather than definitive (Supplemental Table S12).

Comparison-adjusted funnel plots for all four primary networks showed visually symmetric distributions of study-level contrasts, without clear evidence of small-study effects favouring more intensive therapy (Supplemental Figure S3); formal asymmetry tests were not performed due to limited studies per comparison. The proportion of information arising from direct versus indirect evidence is reported in Supplemental Table S13 and the full percentage contribution matrix in Supplemental Figure S4; the quadruple-versus-triple and quadruple-versus-double comparisons were informed entirely by indirect evidence, and quadruple-versus-single/none rested on a single direct study. In an era-stratified ACM analysis (all-measures network, since the HR-only network was too sparse to stratify by era), the magnitude of the triple-therapy association was similar before 2021 (relative effect 0.41; 0.32–0.52) and from 2021 onward (0.57; 0.45–0.72); quadruple estimates were available only from 2021 onward, so that publication era and the availability of the quadruple node are collinear (Supplemental Figure S5).

## 4. Discussion

This systematic review and network meta-analysis of 27 studies enrolling 73,174 patients provides the first network-level synthesis of the clinical outcomes associated with different intensities of GDMT prescribed at hospital discharge in HF. Its most consequential finding is not the direction of the association but the state of the evidence underlying it: of 27 studies addressing this clinically pivotal decision point, only one was a randomized controlled trial, and that trial (STRONG-HF) was conducted before quadruple therapy became the standard of care, testing rapid up-titration intensity rather than the initiation of all four foundational pillars and excluding SGLT2i from its protocol [10]. The comparative evidence on GDMT intensity at discharge is therefore almost entirely observational, and no randomized trial has yet evaluated the safety and efficacy of protocolized quadruple-therapy initiation in the peri-discharge setting. Furthermore, the observational evidence indicates that only about one-third of patients in contemporary SGLT2i-era cohorts are being discharged on all four pillars, highlighting a significant gap despite guideline recommendations.

Within this observational evidence base, we observed a consistent, monotonic gradient in which progressively more complete GDMT was associated with lower relative event rates across all four prespecified outcomes, with quadruple therapy ranking first for each outcome in which it was estimable (P-scores 0.76–0.98); for CV mortality the quadruple node was not estimable in the primary HR-only network, where triple therapy ranked highest. Across outcomes, the quadruple-node comparisons carried the lowest certainty in the network—each informed by at most a single direct study and graded low to very-low confidence, with the headline composite estimate very low—so the first-place ranking of quadruple therapy should be read as a hypothesis-generating signal rather than a precise effect estimate. The directionality was preserved across sensitivity analyses, including the combined HR/RR network, time-window stratification, leave-one-study-out analyses, and an alternative network anchored to the incomplete-therapy node. This gradient is biologically coherent with the complementary mechanisms of the four foundational classes (RASi, beta-blockers, MRAs, and SGLT2i), each modulate distinct but overlapping maladaptive pathways, providing plausibility for additive benefit [2,3]. It is also directionally consistent with prior syntheses conducted predominantly in the ambulatory setting: the component NMA by van Essen et al. and the cross-trial analysis by Vaduganathan et al. both projected substantial incremental benefit from quadruple therapy in chronic HF populations [2,5]. In other words, our observational signal at discharge largely reproduces what randomized outpatient evidence has already established; it does not, on its own, extend the causal knowledge base. Because 26 of 27 studies were nonrandomized and the node-based definition captures the number rather than the specific identity of prescribed pillars, these estimates should be read as associations between greater pharmacological completeness at discharge and better observed outcomes, confounded to an unknown degree by patient selection, rather than as treatment effects or as a ranking of individual molecules.

Several mechanisms other than a true treatment effect plausibly contribute to the observed gradient. Discharge on more complete GDMT is itself a marker of eligibility and clinical stability: patients who tolerate four agents tend to have higher blood pressure and heart-rate reserve, better renal function, less residual congestion, fewer contraindications, and greater engagement with care, whereas sicker or frailer patients are preferentially discharged on fewer agents (confounding by indication and by treatment eligibility). Healthy-adherer bias and institutional effects (higher-performing centres both prescribe more comprehensive therapy and deliver better transitional care) act in the same direction, and our transitivity assessment showed that quadruple-therapy cohorts were, on average, nine years younger and had higher mean eGFR than double-therapy cohorts. The magnitude of association should accordingly not be read as a causal effect.

STRONG-HF remains the conceptual foundation for this question and the only randomized anchor in our synthesis. Its composite benefit (rehospitalisation or death, RR 0.66, 95% CI 0.50–0.86) is directionally and approximately concordant with the triple-therapy association in our network, lending the gradient its only experimental support. However, STRONG-HF tested intensive, protocolised up-titration of three classes (renin–angiotensin modulation, beta-blockade, and mineralocorticoid-receptor antagonism) within a structured high-intensity follow-up programme; it did not include an SGLT2i and enrolled patients across the ejection-fraction spectrum. It therefore does not evaluate contemporary four-pillar therapy. Peri-discharge quadruple initiation is best conceptualised as an extension of the STRONG-HF principle—rapid, supervised optimisation during the vulnerable phase—applied to a regimen that adds a mechanistically distinct fourth class, rather than as a replication of STRONG-HF. Whether the incremental fourth pillar confers benefit beyond intensive triple-therapy up-titration is precisely the question that an adequately powered randomized trial must resolve.

The clinical relevance of these findings must be read together with the well-documented implementation gap at hospital discharge [8]. Real-world registries have consistently shown that a minority of eligible patients leave the hospital on all four foundational classes, and that class-specific prescribing rates vary widely across regions, centers, and patient subgroups [27,28]. Current guideline recommendations favor early initiation of foundational therapies before or at hospital discharge when feasible, while reinforcing that decisions should remain individualized based on hemodynamic status, renal function, potassium balance, and overall tolerability. They also underscore that the incremental contribution of each class should continue to be weighed against patient-level tolerability and shared decision-making, rather than reduced to a numerical target. From a research perspective, our results highlight the need for adequately powered RCTs comparing protocolized quadruple initiation at discharge with standard care, particularly in populations underrepresented in this synthesis, including women, older adults, patients with preserved or mildly reduced ejection fraction, and those from low- and middle-income settings. Implementation-focused studies are similarly needed to test whether structured predischarge optimization pathways, multidisciplinary care models, and digital decision-support tools can drive sustained improvements in GDMT delivery.

**Strengths and Limitations.** To our knowledge, this is the first NMA to synthesise prescription and associated clinical outcomes across the full spectrum of GDMT intensity at hospital discharge, complementing prior component-level syntheses conducted predominantly in the ambulatory setting. The evidence base is contemporary, comprising 27 studies and 73,174 patients across 14 countries and spanning the pre- and post-SGLT2i eras. Consistency was explored through multiple sensitivity analyses, in which the direction and ordering of estimates were generally preserved.

Nonetheless, several limitations should be acknowledged. First, 26 of 27 studies were observational, and residual confounding cannot be excluded; although more than 90% of the comparisons in the HR analysis were derived from multivariable or propensity-score-adjusted models, confounder sets differed across studies, few models explicitly adjusted for contemporaneous disease-severity markers, and none could fully address confounding by indication. Healthier patients (those with better renal function or blood-pressure reserve and those treated in higher-performing systems) may have been more likely to receive quadruple therapy, potentially exaggerating the apparent association between treatment intensity and outcomes; consistent with this, quadruple-therapy cohorts were younger and had higher mean eGFR than lower-intensity cohorts. Second, in the primary HR-only network the formal CINeMA assessment rated confidence as low for eight and very low for thirteen of the 24 comparison–outcome pairs (the three quadruple-therapy CV mortality comparisons were not estimable); no comparison achieved moderate or high confidence. Concerns about heterogeneity were more prominent in the HFH and CV mortality networks than in the ACM and composite networks. Third, the quadruple-therapy node relied on limited direct evidence (one to three studies per outcome), resulting in wide CIs, and was not estimable against single/none therapy for CV mortality in the HR-only network. Fourth, treatment nodes were defined by the number rather than the specific identity or dose of drug classes; within a node, regimens varied, angiotensin-receptor-neprilysin inhibition was used in only a minority of patients in most cohorts, and this within-node heterogeneity, expected to bias individual comparisons toward the null where less efficacious regimens predominate, is reflected in the CINeMA indirectness ratings. Fifth, geographic representation was heavily weighted toward Asia (56% of studies), which may limit generalisability. Sixth, women were under-represented (mean 35.3% female; study-level range 21–76%); because women may derive optimal benefit from neurohormonal antagonists at lower doses and may differ in susceptibility to hypotension, hyperkalaemia, and renal effects, a count-based intensity metric may not map identically onto outcomes across sexes, and study-level reporting was insufficient to perform a sex-stratified meta-analysis. Seventh, node-splitting revealed local inconsistency for the double vs. quadruple contrast in the all-measures ACM network (*p* = 0.034), driven largely by one crude-risk-ratio study; this contrast should be interpreted with caution, although the overall monotonic hierarchy was preserved in the primary HR-only network, across all four outcome networks in the alternative incomplete-anchored parameterization, and in leave-one-out analyses.

## 5. Conclusions

In this network meta-analysis of 27 studies and 73,174 patients with HF, more complete GDMT at hospital discharge was associated with a monotonic gradient of lower event rates across ACM and HFH outcomes, with quadruple therapy ranking first for every endpoint in which it was estimable, but being prescribed suboptimally among contemporary SGLT2i-era cohorts (pooled 34%; range 11.6–55.2%). Certainty of evidence in this meta-analysis was low or very low, reflecting the predominantly observational evidence base, limited direct evidence for several quadruple-therapy comparisons, and evidence of residual confounding. Adequately powered randomized trials of protocolized in-hospital quadruple initiation are therefore the key next step to establish the incremental benefit of this approach and to close the gap between evidence and practice.

## Figures and Tables

**Table 1:** Characteristics of Included Studies.

Study	Country	Design	N	Age, y	Female, %	LVEF, %	GDMT Exposure Definition	Follow-Up, mo	Outcomes
Abe T (2020) [S1]	Japan	Retrospective	885	68.1	28.3	33.5	BB + RASi + MRA	19	ACM, CV mortality
Abe T (2023) [S2]	Japan	Retrospective	1021	77.8	36.3	33.0	BB + RASi	12	ACM
Abe T (2024) [S3]	Japan	Retrospective	160	67.8	21.0	30.5	RASi + BB + MRA	24	CV mortality
Ahn MS (2019) [S4]	South Korea	Prospective	986	68.7	62.6	27.2	RASi + BB + MRA	12	ACM, ACM + HFH, HFH
Akita K (2017) [S5]	Japan	Prospective	773	70.8	36.0	—	BB + RASi	24	HFH
Busson A (2018) [S6]	France	Prospective	624	73.8	35.4	28.2	BB + RASi	12	ACM
Chen YL (2021) [S7]	Taiwan	Prospective	1275	63.1	27.7	28.5	RASi + BB + MRA	12	ACM, CV mortality
Chen J (2025) [S8]	China	Prospective	223	68.8	33.6	32.4	RASi + BB + MRA + SGLT2i	6	ACM + HFH
Dobarro D (2020) [S9]	Spain	Retrospective	280	65.4	26.4	29.8	RASi + BB + MRA	43	ACM
Echeverria LE (2025) [S10]	Colombia	Retrospective	2051	68.0	29.0	30.0	BB + RASi + MRA + SGLT2i	1	ACM, ACM + HFH, HFH
Gilstrap L (2023) [S11]	USA	Retrospective	48,711	79.0	40.6	30.0	BB + RASi	12	ACM
Grewal D (2021) [S12]	USA	Retrospective	1655	63.0	33.0	23.0	BB + RASi + MRA	12	ACM, HFH
Huang CC (2025) [S13]	Taiwan	Retrospective	343	65.1	28.3	28.7	BB + RASi + MRA + SGLT2i	36	ACM, HFH, CV mortality
Jensen H (2025) [S14]	USA	Retrospective	544	59.0	23.7	24.0	BB + RASi + MRA + SGLT2i	3	HFH
Kawakubo Y (2022) [S15]	Japan	Retrospective	1232	72.0	38.0	30.0	BB + RASi + MRA	24	ACM, ACM + HFH, HFH
Mebazaa A (2022) [10]	Multinational (14 countries)	Open-label, parallel-group	1078	63.0	39.0	36.3	BB + RASi + MRA	6	ACM, ACM + HFH, HFH, CV mortality
Miyoshi Y (2026) [S16]	Japan	Prospective	2086	76.5	35.5	34.3	BB + RASi + MRA	12	ACM, ACM + HFH, HFH, CV mortality
Ohata T (2024) [S17]	Japan	Prospective	366	66.5	29.0	—	BB + RASi + MRA	12	ACM + HFH
Oren D (2024) [S18]	USA	Retrospective	603	70.0	37.6	25.2	BB + RASi + MRA + SGLT2i	18	ACM
Severino P (2024) [S19]	Italy	Prospective	278	69.7	76.3	31.4	BB + RASi + MRA + SGLT2i	6	ACM + HFH, HFH, CV mortality
Sung SH (2018) [S20]	Taiwan	Retrospective	423	84.5	21.7	—	RASi + BB + MRA	27	ACM
Takeuchi S (2022) [S21]	Japan	Prospective	1924	73.0	30.5	34.1	BB + RASi	16	ACM, ACM + HFH, HFH
Vicent L (2019) [S22]	Spain	Prospective	566	68.2	33.3	—	RASi + BB + MRA	12	ACM, ACM + HFH, CV mortality
Vorilhon C (2015) [S23]	France	Retrospective	1825	77.2	52.4	—	RASi + BB + MRA	19	ACM
Willeford A (2025) [S24]	USA	Retrospective	2121	59.3	25.6	24.8	BB + RASi + MRA + SGLT2i	1	ACM, ACM + HFH, HFH
Wongsalap Y (2023) [S25]	Thailand	Retrospective	607	64.3	45.5	28.1	BB + RASi + MRA	12	ACM, ACM + HFH, HFH
Yamaguchi T (2018) [S26]	Japan	Prospective	534	73.1	27.1	29.8	BB + RASi	12	ACM, HFH

Abbreviations: ACM = all-cause mortality; CV = cardiovascular; BB = beta-blocker; GDMT = guideline-directed medical therapy; HFH = heart failure hospitalization; LVEF = left ventricular ejection fraction; MRA = mineralocorticoid receptor antagonist; RASi = renin–angiotensin system inhibitor; SGLT2i = sodium–glucose cotransporter 2 inhibitor.

**Table 2:** Primary Network Meta-Analysis Results: All Outcomes, Overall Follow-Up.

Outcome	Comparison	HR [95% CI]	Studies	P-Score	Tau^2^	p-Het
**All-cause mortality**	QT vs. S/N	0.400 [0.091; 1.754]	13	0.760	0.0070	0.194
	TT vs. S/N	0.585 [0.492; 0.696]		0.623		
	DT vs. S/N	0.594 [0.539; 0.655]		0.579		
**Composite ACM + HFH**	QT vs. S/N	0.520 [0.360; 0.751]	4	0.975	0.0000	0.956
	TT vs. S/N	0.721 [0.639; 0.814]		0.601		
	DT vs. S/N	0.756 [0.683; 0.837]		0.424		
**HF hospitalization**	QT vs. S/N	0.638 [0.468; 0.869]	9	0.915	0.0041	0.314
	TT vs. S/N	0.762 [0.664; 0.875]		0.604		
	DT vs. S/N	0.789 [0.705; 0.883]		0.481		
**CV mortality**	QT vs. S/N	Not estimable *	4	—	0.0589	0.138
	TT vs. S/N	0.600 [0.381; 0.944]		0.921		
	DT vs. S/N	0.746 [0.492; 1.130]		0.531		

Values represent network estimates (pooled HRs) from the primary random-effects model restricted to adjusted HRs, using single/none therapy as the reference. * The quadruple node was not estimable against single/none therapy for cardiovascular mortality in the HR-only network; the corresponding all-measures (HR + RR) estimate (0.08, 95% CI 0.02–0.40) is provided in Supplemental Table S7. Abbreviations: ACM = all-cause mortality; CI = confidence interval; CV = cardiovascular; DT = double therapy; HFH = heart failure hospitalization; HR = hazard ratio; QT = quadruple therapy; S/N = single/none therapy; TT = triple therapy; τ^2^ = between-study variance.

**Table 3:** Illustrative Absolute Risk Reduction and Number Needed to Treat (Composite of All-Cause Mortality and HF Hospitalization).

Comparison	Baseline 1-yr Composite Risk	Treated Risk	ARR (95% CI)	NNT (95% CI)
Double vs. single/none	40%	32.0%	8.0% (5.2 to 10.5)	13 (9 to 19)
Triple vs. single/none	40%	30.8%	9.2% (6.0 to 12.2)	11 (8 to 17)

ARR = absolute risk reduction; HR = hazard ratio; NNT = number needed to treat. Additional baseline-risk scenarios are provided in Supplemental Table S10.

## Data Availability

All data analyzed during this study were extracted from the published articles cited in the reference list and the Online Resources. The R code and the structured extraction dataset used for the network meta-analysis are available from the corresponding authors on reasonable request.

## Supplementary Materials

The following supporting information can be downloaded at: https://www.mdpi.com/article/10.3390/jcm15135112/s1, Table S1. PRISMA 2020 checklist.
