# Supplementary material for: GDMT Intensity at Hospital Discharge and Associated Clinical Outcomes in Heart Failure: A Systematic Review and Network Meta-Analysis
Source: J Clin Med. 2026 Jul 1;15(13):5112. doi: 10.3390/jcm15135112 (PMC13363175; doi:10.3390/jcm15135112)
Supplement: Supplementary file 1 [file jcm-15-05112-s001.zip › jcm-4363737-supplementary.pdf]

Supplementary Materials

GDMT Intensity at Hospital Discharge and Associated Clinical Outcomes in Heart Failure: A Systematic Review and Network Meta-Analysis

Supplemental Texts

Supplemental Text S1. Full search strategy for MEDLINE (PubMed).
Supplemental Text S2. Full search strategy for EMBASE (Elsevier).
Supplemental Text S3. Reference list of included studies in the meta-analysis.

Supplemental Tables

Supplemental Table S1. PRISMA 2020 checklist.
Supplemental Table S2. Constituent drug classes and ARNI frequency by treatment node.
Supplemental Table S3. Newcastle–Ottawa Scale quality assessments.
Supplemental Table S4. Master summary of all network meta-analysis estimates vs single/none therapy.
Supplemental Table S5. CINeMA confidence ratings for all primary comparisons.
Supplemental Table S6. CINeMA domain-specific judgments and reasons for downgrading.
Supplemental Table S7. Sensitivity analyses: time-stratified and all-measures vs HR-only results.
Supplemental Table S8. Leave-one-out sensitivity analysis for all-cause mortality.
Supplemental Table S9. Distribution of effect modifiers across treatment-intensity nodes (transitivity).
Supplemental Table S10. Illustrative absolute risk reduction and NNT across baseline-risk scenarios.
Supplemental Table S11. Design-based decomposition of heterogeneity (Q-statistics).
Supplemental Table S12. Alternative network: sensitivity analyses vs incomplete therapy.
Supplemental Table S13. Proportion of direct versus indirect evidence for major comparisons.

Supplemental Figures

Supplemental Figure S1. Characteristics of the 27 included studies.
Supplemental Figure S2. Node-splitting assessment of local inconsistency.
Supplemental Figure S3. Comparison-adjusted funnel plots for all primary outcomes.
Supplemental Figure S4. Percentage contribution matrix (primary HR-only ACM network). (new in R1)
Supplemental Figure S5. Era-stratified all-cause mortality network meta-analysis.

Supplemental Text S1. Full search strategy for MEDLINE (PubMed).

(("Heart Failure"[MeSH Terms] OR "Heart Failure, Systolic"[MeSH Terms] OR "Heart Failure, Diastolic"[MeSH Terms] OR "Ventricular Dysfunction, Left"[MeSH Terms] OR "Cardiomyopathies"[MeSH Terms] OR "heart failure"[tiab] OR "cardiac failure"[tiab] OR "HFrEF"[tiab] OR "HFpEF"[tiab] OR "HFmrEF"[tiab] OR "systolic dysfunction"[tiab] OR "left ventricular dysfunction"[tiab] OR "ventricular failure"[tiab] OR "congestive heart failure"[tiab] OR "CHF"[tiab] OR "decompensated heart failure"[tiab] OR "acute heart failure"[tiab] OR "cardiomyopath*"[tiab]) AND ("Patient Discharge"[MeSH Terms] OR "Hospitalization"[MeSH Terms] OR "Patient Readmission"[MeSH Terms] OR "Aftercare"[MeSH Terms] OR "Transitional Care"[MeSH Terms] OR "discharg*"[tiab] OR "hospital*"[tiab] OR "post-discharg*"[tiab] OR "postdischarg*"[tiab] OR "in-hospital"[tiab] OR "inhospital"[tiab] OR "inpatient*"[tiab] OR "in-patient*"[tiab] OR "transition of care"[tiab] OR "transitional care"[tiab] OR "predischarg*"[tiab] OR "pre-discharg*"[tiab] OR "index admission"[tiab] OR "index hospitalization"[tiab]) AND ("Drug Therapy, Combination"[MeSH Terms] OR "Angiotensin Receptor Antagonists"[MeSH Terms] OR "Neprilysin"[MeSH Terms] OR "Sodium-Glucose Transporter 2 Inhibitors"[MeSH Terms] OR "Adrenergic beta-Antagonists"[MeSH Terms] OR "Mineralocorticoid Receptor Antagonists"[MeSH Terms] OR "quadruple therap*"[tiab] OR "four-pillar therap*"[tiab] OR "comprehensive medical therap*"[tiab] OR "guideline-directed medical therap*"[tiab] OR "guideline directed medical therap*"[tiab] OR "GDMT"[tiab] OR "disease-modifying therap*"[tiab] OR "combination therap*"[tiab] OR "combination pharmacotherap*"[tiab] OR "multi-drug

therap*"[tiab] OR "multidrug therap*"[tiab] OR "angiotensin receptor neprilysin inhibitor*"[tiab] OR "ARNI"[tiab] OR "ARNIs"[tiab] OR "sacubitril-valsartan"[tiab] OR "sacubitril valsartan"[tiab] OR "LCZ696"[tiab] OR "Entresto"[tiab] OR "neprilysin inhibitor*"[tiab] OR "sodium-glucose cotransporter 2 inhibitor*"[tiab] OR "sodium glucose cotransporter 2 inhibitor*"[tiab] OR "SGLT2 inhibitor*"[tiab] OR "SGLT-2 inhibitor*"[tiab] OR "gliflozin*"[tiab] OR "empagliflozin"[tiab] OR "dapagliflozin"[tiab] OR "sotagliflozin"[tiab] OR "canagliflozin"[tiab] OR "beta-blocker*"[tiab] OR "beta blocker*"[tiab] OR "beta-adrenergic blocker*"[tiab] OR "beta adrenergic antagonist*"[tiab] OR "bisoprolol"[tiab] OR "carvedilol"[tiab] OR "metoprolol succinate"[tiab] OR "nebivolol"[tiab] OR "mineralocorticoid receptor antagonist*"[tiab] OR "MRA"[tiab] OR "MRAs"[tiab] OR "aldosterone antagonist*"[tiab] OR "aldosterone blocker*"[tiab] OR "spironolactone"[tiab] OR "eplerenone"[tiab] OR "finerenone"[tiab]) AND ("early initiat*"[tiab] OR "simultaneous initiat*"[tiab] OR "rapid initiat*"[tiab] OR "early uptitrat*"[tiab] OR "rapid uptitrat*"[tiab] OR "early up-titrat*"[tiab] OR "rapid up-titrat*"[tiab] OR "titrat*"[tiab] OR "uptitrat*"[tiab] OR "up-titrat*"[tiab] OR "optimiz*"[tiab] OR "optimis*"[tiab] OR "intensif*"[tiab] OR "early prescri*"[tiab] OR "predischarge initiat*"[tiab] OR "pre-discharge initiat*"[tiab] OR "in-hospital initiat*"[tiab] OR "before discharge"[tiab] OR "prior to discharge"[tiab] OR "at discharge"[tiab] OR "upon discharge"[tiab] OR "initiat* before discharg*"[tiab] OR "initiat* at discharg*"[tiab] OR "prescri* at discharg*"[tiab] OR "simultaneous prescri*"[tiab] OR "concurrent initiat*"[tiab] OR "comprehensive initiat*"[tiab] OR "early implementat*"[tiab]))

Supplemental Text S2. Full search strategy for EMBASE (Elsevier).

('heart failure'/exp OR 'left ventricle dysfunction' OR 'cardiomyopathy'/exp OR 'heart failure':ti,ab OR 'cardiac failure':ti,ab OR 'hfref':ti,ab OR 'hfpef':ti,ab OR 'hfmref':ti,ab OR 'systolic dysfunction':ti,ab OR 'left ventricular dysfunction':ti,ab OR 'ventricular failure':ti,ab OR 'congestive heart failure':ti,ab OR 'chf':ti,ab OR 'decompensated heart failure':ti,ab OR 'acute heart failure':ti,ab OR cardiomyopath*:ti,ab) AND ('hospital discharge'/exp

OR 'hospitalization'/exp OR 'hospital readmission'/exp OR 'aftercare'/exp OR 'transitional care'/exp OR 'hospital discharge':ti,ab OR 'patient discharge':ti,ab OR 'discharge planning':ti,ab OR discharged:ti,ab OR hospitali?ation:ti,ab OR hospitali?ed:ti,ab OR hospitali?e:ti,ab OR 'hospital admission':ti,ab OR 'hospital stay':ti,ab OR 'post-discharge':ti,ab OR postdischarg*:ti,ab OR 'in-hospital':ti,ab OR inhospital:ti,ab OR inpatient*:ti,ab OR 'in-patient*':ti,ab OR 'transition of care':ti,ab OR 'transitional care':ti,ab OR predischarg*:ti,ab OR 'pre-discharge':ti,ab OR 'index admission':ti,ab OR 'index hospitalization':ti,ab) AND ('combination drug therapy'/exp OR 'angiotensin receptor antagonist'/exp OR 'neprilysin'/exp OR 'sacubitril plus valsartan'/exp OR 'sodium glucose cotransporter 2 inhibitor'/exp OR 'beta adrenergic receptor blocking agent'/exp OR 'mineralocorticoid receptor antagonist'/exp OR 'quadruple therapy':ti,ab OR 'four-pillar therapy':ti,ab OR 'comprehensive medical therapy':ti,ab OR 'guideline-directed medical therapy':ti,ab OR 'guideline directed medical therapy':ti,ab OR 'gdmt':ti,ab OR 'disease-modifying therapy':ti,ab OR 'combination pharmacotherapy':ti,ab OR 'multi-drug therapy':ti,ab OR 'multidrug therapy':ti,ab OR 'angiotensin receptor neprilysin inhibitor*':ti,ab OR 'arni':ti,ab OR 'arnis':ti,ab OR 'sacubitril-valsartan':ti,ab OR 'sacubitril valsartan':ti,ab OR 'lcz696':ti,ab OR 'entresto':ti,ab OR 'neprilysin inhibitor*':ti,ab OR 'sodium-glucose cotransporter 2 inhibitor*':ti,ab OR 'sodium glucose cotransporter 2 inhibitor*':ti,ab OR 'sglt2 inhibitor*':ti,ab OR 'sglt-2 inhibitor*':ti,ab OR gliflozin*:ti,ab OR empagliflozin:ti,ab OR dapagliflozin:ti,ab OR sotagliflozin:ti,ab OR canagliflozin:ti,ab OR 'beta-blocker*':ti,ab OR 'beta blocker*':ti,ab OR 'beta-adrenergic blocker*':ti,ab OR 'beta adrenergic antagonist*':ti,ab OR bisoprolol:ti,ab OR carvedilol:ti,ab OR 'metoprolol succinate':ti,ab OR nebivolol:ti,ab OR 'mineralocorticoid receptor antagonist*':ti,ab OR 'mra':ti,ab OR 'mras':ti,ab OR 'aldosterone antagonist*':ti,ab OR 'aldosterone blocker*':ti,ab OR spironolactone:ti,ab OR eplerenone:ti,ab OR finerenone:ti,ab) AND ('early initiat*':ti,ab OR 'simultaneous initiat*':ti,ab OR 'rapid initiat*':ti,ab OR 'early uptitrat*':ti,ab OR 'rapid uptitrat*':ti,ab OR 'early up-titrat*':ti,ab OR 'rapid up-titrat*':ti,ab OR uptitrat*:ti,ab OR 'up-titrat*':ti,ab OR (((optimiz* OR optimis*) NEAR/3 (therap* OR treatment* OR medication* OR dose* OR dosing OR pharmacotherap*)):ti,ab) OR ((intensif* NEAR/3 (therap* OR treatment* OR medication* OR pharmacotherap*)):ti,ab) OR 'early prescri*':ti,ab OR

'predischarge initiat*':ti,ab OR 'pre-discharge initiat*':ti,ab OR 'in-hospital initiat*':ti,ab OR 'before discharge':ti,ab OR 'prior to discharge':ti,ab OR 'at discharge':ti,ab OR 'upon discharge':ti,ab OR 'initiat* before discharg*':ti,ab OR 'initiat* at discharg*':ti,ab OR 'prescri* at discharg*':ti,ab OR 'simultaneous prescri*':ti,ab OR 'concurrent initiat*':ti,ab OR 'comprehensive initiat*':ti,ab OR 'early implementat*':ti,ab)

Supplemental Text S3. Reference list of included studies in the meta-analysis.

S1. Abe T, Jujo K, Kametani M, et al. Prognostic impact of additional mineralocorticoid receptor antagonists in octogenarian heart failure patients. ESC Heart Fail 2020;7:2711–2724.

S2. Abe T, Jujo K, Maeda D, et al. The interaction between physical frailty and prognostic impact of heart failure medication in elderly patients. ESC Heart Fail 2023;10:1698–1705.

S3. Abe T, Shirotani S, Takada T, et al. Additional mineralocorticoid receptor antagonists in heart failure underdosed with guideline-directed medication. J Renin Angiotensin Aldosterone Syst 2024;25:14703203241306037.

S4. Ahn MS, Yoo BS, Yoon J, et al. Prognostic effect of guideline-directed therapy is more noticeable early in the course of heart failure. J Korean Med Sci 2019;34:e133. 16

S5. Akita K, Kohno T, Kohsaka S, et al. Current use of guideline-based medical therapy in elderly patients admitted with acute heart failure with reduced ejection fraction and its impact on event-free survival. Int J Cardiol 2017;235:162–168. 17

S6. Busson A, Thilly N, Laborde-Castérot H, et al. Effectiveness of guideline-consistent heart failure drug prescriptions at hospital discharge on 1-year mortality: results from the EPICAL2 cohort study. Eur J Intern Med 2018;51:53–60.

S7. Chen J, Xiao R, Gao L, et al. Real-world challenges for guideline-directed medical therapy intolerance in heart failure: a single-center prospective cohort study. Int J Cardiol 2025;434:133367.

S8. Chen Y-L, Hang C-L, Su C-H, et al. Feature and impact of guideline-directed medication prescriptions for heart failure with reduced ejection fraction accompanied by chronic kidney disease. Int J Med Sci 2021;18:2570–2580.

S9. Dobarro D, Melendo-Viu M, Raposeiras-Roubín S, et al. Triple neurohormonal blockade in de novo heart failure with reduced ejection fraction during index hospitalization. REC: CardioClinics 2020;55:79–85.

S10. Echeverría LE, Rojas LZ, Serrano-García AY, et al. Early prescription of quadruple therapy in acute decompensated heart failure with reduced ejection fraction: a propensity score-matched analysis. ESC Heart Fail 2025;12:2814–2826.

S11. Gilstrap L, Solomon N, Chiswell K, et al. The association between beta-blocker and renin-angiotensin system inhibitor use after heart failure with reduced ejection fraction hospitalization and outcomes in older patients. J Card Fail 2023;29:434–444.

S12. Grewal D, Partow-Navid R, Garcia D, et al. Role of guideline directed medical therapy doses and optimization in patients hospitalized with decompensated systolic heart failure. Am J Cardiol 2021;151:64–69.

S13. Huang C-C, Tzeng B-H, Tsai H-Y, et al. Early initiation of guideline-directed medical therapy improves outcomes in heart failure with reduced ejection fraction patients without significant risk of nephrotoxicity. Acta Cardiol Sin 2025;41:510–520.

S14. Jensen H, Staskauskas M, Strome S, et al. Assessment of guideline-directed medical therapy optimization scores and readmission risk in heart failure with reduced ejection fraction. Ann Pharmacother 2025;10600280251387249.

S15. Kawakubo Y, Shiraishi Y, Kohsaka S, et al. Potential association with malnutrition and allocation of combination medical therapies in hospitalized heart failure patients with reduced ejection fraction. Sci Rep 2022;12:8318.

S16. Miyoshi Y, Kato T, Morimoto T, et al. Prevalence and clinical significance of guideline-directed medical therapy in acute heart failure with reduced or mildly reduced ejection fraction. Sci Rep 2026;16:5116.

S17. Ohata T, Niimi N, Shiraishi Y, et al. Initiation and up-titration of guideline-based medications in hospitalized acute heart failure patients—a report from the West Tokyo Heart Failure Registry. Circ J 2023;88:22–30.

S18. Oren D, Elsaygh J, Moeller CM, et al. Utility of guideline-directed therapy on mortality and readmissions in socioeconomically disadvantaged heart failure patients. Cardiol Rev 2024; doi:10.1097/CRD.0000000000000823.

S19. Severino P, D'Amato A, Prosperi S, et al. Strategy for an early simultaneous introduction of four-pillars of heart failure therapy: results from a single center experience. Am J Cardiovasc Drugs 2024;24:663–671.

S20. Sung S-H, Wang T-J, Cheng H-M, et al. Clinical characteristics and outcomes in the very elderly patients hospitalized for acute heart failure: importance of pharmacologic guideline adherence. Sci Rep 2018;8:14270.

S21. Takeuchi S, Kohno T, Goda A, et al. Multimorbidity, guideline-directed medical therapies, and associated outcomes among hospitalized heart failure patients. ESC Heart Fail 2022;9:2500–2510.

S22. Vicent L, Cinca J, Vazquez-García R, et al. Discharge treatment with angiotensin-converting enzyme inhibitor/angiotensin receptor blocker after a heart failure hospitalisation is associated with a better prognosis irrespective of left ventricular ejection fraction. Intern Med J 2019;49:1505–1513.

S23. Vorilhon C, Chenaf C, Mulliez A, et al. Heart failure prognosis and management in over-80-year-old patients: data from a French national observational retrospective cohort. Eur J Clin Pharmacol 2015;71:251–260.

S24. Willeford A, Greenberg B, Yousif Z. Additive benefit of guideline-directed medical therapies at discharge in reducing 30-day readmissions in heart failure. JACC Adv 2026;5:102411.

S25. Wongsalap Y, Poolpun D, Keawhai K, et al. Pharmacotherapy treatment patterns at hospital discharge and clinical outcomes among patients with heart failure with reduced ejection fraction. Chronic Dis Transl Med 2023;9:154–163.

S26. Yamaguchi T, Kitai T, Miyamoto T, et al. Effect of optimizing guideline-directed medical therapy before discharge on mortality and heart failure readmission in patients hospitalized with heart failure with reduced ejection fraction. Am J Cardiol 2018;121:969–974.

SUPPLEMENTAL TABLES

Supplemental Table S1. PRISMA 2020 Checklist

Section and Topic	Item #	Checklist item	Location where item is reported
**TITLE**			
Title	1	Identify the report as a systematic review.	p. 1.
**ABSTRACT**			
Abstract	2	See the PRISMA 2020 for Abstracts checklist.	Abstract, pp. 1-2.
**INTRODUCTION**			
Rationale	3	Describe the rationale for the review in the context of existing knowledge.	pp. 2-3.
Objectives	4	Provide an explicit statement of the objective(s) or question(s) the review addresses.	p. 1; p. 2.
**METHODS**			
Eligibility criteria	5	Specify the inclusion and exclusion criteria for the review and how studies were grouped for the syntheses.	p. 3; Figure 1, p. 7.
Information sources	6	Specify all databases, registers, websites, organisations, reference lists and other sources searched or consulted to identify studies. Specify the date when each source was last searched or consulted.	p. 3.
Search strategy	7	Present the full search strategies for all databases, registers and websites, including any filters and limits used.	p. 3; Supplemental Texts S1-S2.
Selection process	8	Specify the methods used to decide whether a study met the inclusion criteria of the review, including how many reviewers screened each record and each report retrieved, whether they worked independently, and if applicable, details of automation tools used in the process.	p. 3; Figure 1, p. 7.
Data collection process	9	Specify the methods used to collect data from reports, including how many reviewers collected data from each report, whether they worked independently, any processes for obtaining or confirming data from study investigators, and if applicable, details of automation tools used in the process.	p. 3; author contributions, p. 16.
Data items	10a	List and define all outcomes for which data were sought. Specify whether all results that were compatible with each outcome domain in each study were sought (e.g. for all measures, time points, analyses), and if not, the methods used to decide which results to collect.	p. 3.
	10b	List and define all other variables for which data were sought (e.g. participant and intervention characteristics, funding sources). Describe any assumptions made about any missing or unclear information.	p. 3
Study risk of bias assessment	11	Specify the methods used to assess risk of bias in the included studies, including details of the tool(s) used, how many reviewers assessed each study and whether they worked independently, and if applicable, details of automation tools used in the process.	pp. 4-5; author contributions, p. 16; Supplemental Table S3.
Effect measures	12	Specify for each outcome the effect measure(s) (e.g. risk ratio, mean difference) used in the synthesis or presentation of results.	p. 4.
Synthesis methods	13a	Describe the processes used to decide which studies were eligible for each synthesis (e.g. tabulating the study intervention characteristics and comparing against the planned groups for each synthesis (item #5)).	pp. 3-4.
	13b	Describe any methods required to prepare the data for presentation or synthesis, such as handling of missing summary statistics, or data conversions.	pp. 3-4.
	13c	Describe any methods used to tabulate or visually display results of individual studies and syntheses.	p. 4.
	13d	Describe any methods used to synthesize results and provide a rationale for the choice(s). If meta-analysis was performed, describe the model(s), method(s) to identify the presence and extent of statistical heterogeneity, and software package(s) used.	p. 4.
	13e	Describe any methods used to explore possible causes of heterogeneity among study results (e.g. subgroup analysis, meta-regression).	pp. 5-6.
	13f	Describe any sensitivity analyses conducted to assess robustness of the synthesized results.	pp. 5-6.
Reporting bias	14	Describe any methods used to assess risk of bias due to missing results in a synthesis (arising from reporting	pp. 4-6.

Section and Topic	Item #	Checklist item	Location where item is reported
assessment		biases).	
Certainty assessment	15	Describe any methods used to assess certainty (or confidence) in the body of evidence for an outcome.	pp. 4-6.
**RESULTS**			
Study selection	16a	Describe the results of the search and selection process, from the number of records identified in the search to the number of studies included in the review, ideally using a flow diagram.	pp. 5-6; Figure 1, p. 7.
	16b	Cite studies that might appear to meet the inclusion criteria, but which were excluded, and explain why they were excluded.	Figure 1, p. 5.
Study characteristics	17	Cite each included study and present its characteristics.	pp. 6-8; Table 1, pp. 7-8; Supplemental Text S3.
Risk of bias in studies	18	Present assessments of risk of bias for each included study.	pp. 5-7; Supplemental Tables S3 and S5-S6.
Results of individual studies	19	For all outcomes, present, for each study: (a) summary statistics for each group (where appropriate) and (b) an effect estimate and its precision (e.g. confidence/credible interval), ideally using structured tables or plots.	Table 1, pp. 7-8; Figure 2/Table 2, pp. 9-10; Supplemental Table S4.
Results of syntheses	20a	For each synthesis, briefly summarise the characteristics and risk of bias among contributing studies.	pp. 9-13; Table 1, pp. 5-8; Supplemental Tables S3 and S5-S6.
	20b	Present results of all statistical syntheses conducted. If meta-analysis was done, present for each the summary estimate and its precision (e.g. confidence/credible interval) and measures of statistical heterogeneity. If comparing groups, describe the direction of the effect.	pp. 7-13; Figures 2-4, pp. 9-12; Tables 2-3; Supplemental Tables S7-S12.
	20c	Present results of all investigations of possible causes of heterogeneity among study results.	pp. 12-13; Supplemental Table S9 and Figure S5.
	20d	Present results of all sensitivity analyses conducted to assess the robustness of the synthesized results.	pp. 12-13; Supplemental Tables S7-S8 and S10-S12.
Reporting biases	21	Present assessments of risk of bias due to missing results (arising from reporting biases) for each synthesis assessed.	pp. 12-13; Supplemental Figure S3.
Certainty of evidence	22	Present assessments of certainty (or confidence) in the body of evidence for each outcome assessed.	pp. 9-13; p. 16; Supplemental Tables S5-S6.
**DISCUSSION**			
Discussion	23a	Provide a general interpretation of the results in the context of other evidence.	pp. 13-15.
	23b	Discuss any limitations of the evidence included in the review.	pp. 15-16.
	23c	Discuss any limitations of the review processes used.	pp. 15-16.
	23d	Discuss implications of the results for practice, policy, and future research.	pp. 15-16.
**OTHER INFORMATION**			
Registration and protocol	24a	Provide registration information for the review, including register name and registration number, or state that the review was not registered.	p. 3 (PROSPERO CRD420261352137).
	24b	Indicate where the review protocol can be accessed, or state that a protocol was not prepared.	p. 3 (PROSPERO registration record).
	24c	Describe and explain any amendments to information provided at registration or in the protocol.	Not reported; no amendments described.
Support	25	Describe sources of financial or non-financial support for the review, and the role of the funders or sponsors in the review.	p. 16.
Competing interests	26	Declare any competing interests of review authors.	p. 16.
Availability of data, code and other materials	27	Report which of the following are publicly available and where they can be found: template data collection forms; data extracted from included studies; data used for all analyses; analytic code; any other materials used in the review.	p. 16.

Supplemental Table S2. Constituent Drug Classes and ARNI Frequency by Treatment Node

Study	Node	BB, %	RASi, %	ARNI, %	MRA, %	SGLT2i, %
Abe T 2020	Triple	100	100	0	100	0
Abe T 2020	Double	100	100	0	0	0
Abe T 2020	Single/None	26	40	0	0	0
Ahn MS	Triple	100	100	0	100	0
Ahn MS	Double	56	85	0	58	0
Ahn MS	Single/None	16	20	0	12	0
Chen J	Incomplete	31	22		15	16
Echeverria LE	Incomplete	91	81		78	74
Gilstrap L	Double	100	100			
Grewal D	Double	73	56		14	
Huang CC	Triple	92	89		97	21
Huang CC	Double	83	52		52	14
Huang CC	Single/None	60	7		20	3
Huang CC	Incomplete	84	65		71	16
Mebazaa A	Triple	98	98		98	0
Mebazaa A	Incomplete	39	64		97	
Miyoshi Y	Triple	100	100	0	100	0
Miyoshi Y	Double	88	70	0	42	0
Miyoshi Y	Single/None	56	25	0	18	0
Miyoshi Y	Incomplete	66	46		29	0
Willeford A	Triple	82	85	43	77	57
Willeford A	Double	67	58	28	35	39
Willeford A	Single/None	53	22	11	14	11
Yamaguchi T	Double	100	100	0	57	0
Yamaguchi T	Single/None	68	32	0	50	0

Discharge prescription proportions by treatment node for the subset of studies reporting node-level composition; blank cells indicate the class proportion was not reported. ARNI use was reported in a minority of cohorts (e.g., Willeford 2025: 43% triple, 28% double, 11% single/none node); in nine of 27 studies the renin–angiotensin component could include ARNI. BB = beta-blocker; MRA = mineralocorticoid receptor antagonist; RASi = renin–angiotensin system inhibitor; SGLT2i = sodium-glucose cotransporter 2 inhibitor.

Supplemental Table S3. Newcastle-Ottawa Scale quality assessments for all 26 observational studies.

Study	S1	S2	S3	S4	C1	O1	O2	O3	Total

	Representativeness of exposed cohort	*Selection of non-exposed cohort*	*Ascertainment of exposure*	*Outcome not present at start*	*Comparability of cohorts*	*Assessment of outcome*	*Follow-up long enough*	*Adequacy of follow-up*	
Abe 2020	–	★	★	★	★★	–	★	★	**7**
Abe 2023	★	★	–	–	★	–	★	★	**5**
Abe 2024	★	★	–	★	★★	–	★	–	**6**
Ahn 2019	★	★	★	★	★★	★	★	★	**9**
Akita 2017	★	★	★	★	★★	★	★	★	**9**
Busson 2018	★	★	★	★	★★	★	★	★	**9**
Chen 2021	★	★	★	★	★	–	★	★	**8**
Chen 2025	★	★	★	★	–	–	★	★	**6**

Dobarro 2020	★	★	★	★	★★	—	★	—	**7**
Echeverria 2025	★	★	★	★	★★	★	★	★	**9**
Gilstrap 2023	★	★	★	★	★★	★	★	★	**9**
Grewal 2021	—	★	★	★	★	★	★	—	**6**
Huang 2025	—	★	★	★	★	★	★	★	**7**
Jensen 2025	★	★	★	★	★★	—	★	—	**7**
Kawakubo 2022	★	★	★	★	★★	★	★	★	**9**
Miyoshi 2026	★	★	★	★	★★	★	★	—	**8**
Ohata 2024	★	★	★	★	★★	★	★	—	**8**

Oren 2024	★	★	★	★	★	—	★	—	**6**
Severino 2024	★	★	—	★	★★	—	★	—	**6**
Sung 2018	★	★	★	—	★★	★	★	—	**7**
Takeuchi 2022	★	★	★	★	★★	★	★	—	**8**
Vicent 2019	★	★	★	★	★	—	★	★	**8**
Vorilhon 2015	★	★	★	★	★	★	★	★	**8**
Willeford 2025	★	★	★	★	★★	—	★	—	**7**
Wongsalap 2023	★	★	★	★	★★	—	★	—	**7**
Yamaguchi 2018	★	★	★	★	★★	★	★	—	**8**

Supplemental Table S4. Master Summary of All Network Meta-Analysis Estimates vs Single/None Therapy

Outcome	Time Window	Measures	Comparison	Effect (95% CI)	k	n	Rows	τ^2	p-het
**Composite ACM + HFH**	30–90 days	HR only	Triple vs Single/None	0.680 [0.515; 0.898]	1	3	3	—	—
			Double vs Single/None	0.800 [0.632; 1.013]	1	3	3	—	—
			Quadruple vs Single/None	0.520 [0.360; 0.751]	1	3	3	—	—
		All measures	Triple vs Single/None	0.680 [0.515; 0.898]	1	3	3	—	—
			Double vs Single/None	0.800 [0.632; 1.013]	1	3	3	—	—
			Quadruple vs Single/None	0.520 [0.360; 0.751]	1	3	3	—	—
	6–12 months	HR only	Triple vs Single/None	0.750 [0.602; 0.934]	1	2	2	—	—
			Double vs Single/None	0.773 [0.573; 1.044]	1	2	2	—	—
		All measures	Triple vs Single/None	0.667 [0.588; 0.756]	3	6	6	0.0000	0.493
			Double vs Single/None	0.709 [0.613; 0.820]	3	6	6	0.0000	0.493
	>12 months	HR only	Triple vs Single/None	0.700 [0.544; 0.900]	2	3	3	0.0000	0.498
			Double vs Single/None	0.749 [0.660; 0.849]	2	3	3	0.0000	0.498
		All measures	Triple vs Single/None	0.700 [0.544; 0.900]	2	3	3	0.0000	0.498
			Double vs Single/None	0.749 [0.660; 0.849]	2	3	3	0.0000	0.498
	Overall	HR only	Triple vs Single/None	0.721 [0.639; 0.814]	4	8	8	0.0000	0.956
			Double vs Single/None	0.756 [0.683; 0.837]	4	8	8	0.0000	0.956
			Quadruple vs Single/None	0.520 [0.360; 0.751]	4	8	8	0.0000	0.956
		All measures	Triple vs Single/None	0.681 [0.615; 0.753]	6	12	12	0.0000	0.859
			Double vs Single/None	0.742 [0.679; 0.809]	6	12	12	0.0000	0.859
			Quadruple vs Single/None	0.520 [0.360; 0.751]	6	12	12	0.0000	0.859
**HF hospitalization**	30–90 days	HR only	Triple vs Single/None	0.780 [0.555; 1.096]	3	8	8	0.0293	0.167

			Double vs Single/None	0.768 [0.576; 1.025]	3	8	8	0.0293	0.167
			Quadruple vs Single/None	0.635 [0.432; 0.936]	3	8	8	0.0293	0.167
		All measures	Triple vs Single/None	0.780 [0.555; 1.096]	3	8	8	0.0293	0.167
			Double vs Single/None	0.768 [0.576; 1.025]	3	8	8	0.0293	0.167
			Quadruple vs Single/None	0.635 [0.432; 0.936]	3	8	8	0.0293	0.167
	6–12 months	HR only	Triple vs Single/None	0.869 [0.705; 1.072]	3	5	5	0.0000	0.877
			Double vs Single/None	1.007 [0.786; 1.290]	3	5	5	0.0000	0.877
		All measures	Triple vs Single/None	0.761 [0.540; 1.072]	4	6	11	0.0537	0.089
			Double vs Single/None	0.986 [0.684; 1.422]	4	6	11	0.0537	0.089
			Quadruple vs Single/None	0.354 [0.159; 0.785]	4	6	11	0.0537	0.089
	>12 months	HR only	Triple vs Single/None	0.650 [0.488; 0.866]	3	4	4	0.0000	0.642
			Double vs Single/None	0.731 [0.638; 0.839]	3	4	4	0.0000	0.642
		All measures	Triple vs Single/None	0.650 [0.488; 0.866]	3	4	4	0.0000	0.642
			Double vs Single/None	0.731 [0.638; 0.839]	3	4	4	0.0000	0.642
	Overall	HR only	Triple vs Single/None	0.762 [0.664; 0.875]	9	17	17	0.0041	0.314
			Double vs Single/None	0.789 [0.705; 0.883]	9	17	17	0.0041	0.314
			Quadruple vs Single/None	0.638 [0.468; 0.869]	9	17	17	0.0041	0.314
		All measures	Triple vs Single/None	0.734 [0.616; 0.873]	10	18	23	0.0210	0.049
			Double vs Single/None	0.802 [0.693; 0.928]	10	18	23	0.0210	0.049
			Quadruple vs Single/None	0.552 [0.399; 0.764]	10	18	23	0.0210	0.049
**CV mortality**	6–12 months	HR only	Triple vs Single/None	0.665 [0.487; 0.906]	2	3	3	0.0000	0.634
			Double vs Single/None	0.624 [0.455; 0.858]	2	3	3	0.0000	0.634
		All measures	Triple vs Single/None	0.537 [0.372; 0.776]	3	5	5	0.0402	0.159

			Double vs Single/None	0.562 [0.388; 0.815]	3	5	5	0.0402	0.159
	>12 months	HR only	Triple vs Single/None	0.383 [0.125; 1.174]	2	3	3	0.0635	0.260
			Double vs Single/None	0.850 [0.371; 1.949]	2	3	3	0.0635	0.260
		All measures	Triple vs Single/None	0.292 [0.144; 0.591]	3	4	9	0.0000	0.535
			Double vs Single/None	0.700 [0.412; 1.189]	3	4	9	0.0000	0.535
			Quadruple vs Single/None	0.073 [0.015; 0.349]	3	4	9	0.0000	0.535
	Overall	HR only	Triple vs Single/None	0.600 [0.381; 0.944]	4	6	6	0.0589	0.138
			Double vs Single/None	0.746 [0.492; 1.130]	4	6	6	0.0589	0.138
		All measures	Triple vs Single/None	0.448 [0.305; 0.658]	6	9	14	0.0761	0.069
			Double vs Single/None	0.642 [0.450; 0.915]	6	9	14	0.0761	0.069
			Quadruple vs Single/None	0.081 [0.016; 0.399]	6	9	14	0.0761	0.069
**All-cause mortality**	6–12 months	HR only	Triple vs Single/None	0.600 [0.492; 0.730]	8	11	11	0.0006	0.418
			Double vs Single/None	0.550 [0.506; 0.599]	8	11	11	0.0006	0.418
		All measures	Triple vs Single/None	0.495 [0.403; 0.608]	10	14	19	0.0150	0.113
			Double vs Single/None	0.539 [0.470; 0.617]	10	14	19	0.0150	0.113
			Quadruple vs Single/None	0.081 [0.033; 0.198]	10	14	19	0.0150	0.113
	>12 months	HR only	Triple vs Single/None	0.565 [0.435; 0.734]	5	9	9	0.0000	0.777
			Double vs Single/None	0.696 [0.609; 0.795]	5	9	9	0.0000	0.777
			Quadruple vs Single/None	0.400 [0.092; 1.738]	5	9	9	0.0000	0.777
		All measures	Triple vs Single/None	0.565 [0.435; 0.734]	5	9	9	0.0000	0.777
			Double vs Single/None	0.696 [0.609; 0.795]	5	9	9	0.0000	0.777
			Quadruple vs Single/None	0.400 [0.092; 1.738]	5	9	9	0.0000	0.777
	Overall	HR only	Triple vs Single/None	0.585 [0.492; 0.696]	13	20	20	0.0070	0.194

			Double vs Single/None	0.594 [0.539; 0.655]	13	20	20	0.0070	0.194
			Quadruple vs Single/None	0.400 [0.091; 1.754]	13	20	20	0.0070	0.194
		All measures	Triple vs Single/None	0.519 [0.437; 0.616]	15	23	28	0.0168	0.041
			Double vs Single/None	0.594 [0.531; 0.664]	15	23	28	0.0168	0.041
			Quadruple vs Single/None	0.129 [0.060; 0.278]	15	23	28	0.0168	0.041

Supplemental Table S5. CINeMA Confidence Ratings for All Primary Network Meta-Analysis Comparisons

Outcome	Comparison	HR (95% CI)	k	WSB	RB	IND	IMP	HET	INC	Confidence
All-Cause Mortality	QT vs S/N	0.40 [0.09, 1.75]	1	●	◒	◒	●	○	○	Very low
	TT vs S/N	0.59 [0.49, 0.70]	6	●	◒	◒	○	○	○	Low
	DT vs S/N	0.59 [0.54, 0.66]	11	●	◒	◒	○	○	○	Low
	QT vs TT	0.68 [0.15, 3.03]	0	●	◒	◒	●	○	○	Very low
	QT vs DT	0.67 [0.15, 2.96]	0	●	◒	◒	●	○	○	Very low
	TT vs DT	0.99 [0.82, 1.18]	2	●	◒	◒	●	○	○	Very low
Composite ACM + HFH	QT vs S/N	0.52 [0.36, 0.75]	1	●	◒	◒	○	○	○	Very low
	TT vs S/N	0.72 [0.64, 0.81]	3	●	◒	◒	○	○	○	Low
	DT vs S/N	0.76 [0.68, 0.84]	3	●	◒	◒	○	○	○	Low
	QT vs TT	0.72 [0.49, 1.06]	0	●	◒	◒	●	○	○	Very low
	QT vs DT	0.69 [0.47, 1.01]	0	●	◒	◒	◒	○	○	Low
	TT vs DT	0.95 [0.83, 1.09]	1	●	◒	◒	◒	○	○	Low
HF Hospitalization	QT vs S/N	0.64 [0.47, 0.87]	2	●	◒	◒	○	○	○	Low
	TT vs S/N	0.76 [0.66, 0.88]	6	●	◒	◒	○	◒	○	Very low
	DT vs S/N	0.79 [0.71, 0.88]	8	●	◒	◒	○	●	○	Very low
	QT vs TT	0.84 [0.60, 1.17]	0	●	◒	◒	●	◒	○	Very low
	QT vs DT	0.81 [0.58, 1.12]	0	●	◒	◒	●	●	○	Very low
	TT vs DT	0.97 [0.82, 1.13]	1	●	◒	◒	●	○	○	Very low
CV Mortality	QT vs S/N	Not estimable (HR-only)	—	—	—	—	—	—	—	Not estimable
	TT vs S/N	0.60 [0.38, 0.94]	1	●	◒	◒	◒	○	○	Low

	DT vs S/N	0.75 [0.49, 1.13]	2	●	◒	◒	●	●	○	Very low
	QT vs TT	Not estimable (HR-only)	—	—	—	—	—	—	—	Not estimable
	QT vs DT	Not estimable (HR-only)	—	—	—	—	—	—	—	Not estimable
	TT vs DT	0.80 [0.54, 1.20]	3	●	◒	◒	●	◒	○	Very low

Domain ratings: ○ No concerns ◒ Some concerns ● Major concerns

For CINeMA, all observational studies were judged to have high within-study bias because the nonrandomized allocation of discharge GDMT intensity was inherently vulnerable to residual confounding by indication, treatment-selection bias, and incomplete control of clinical stability and treatment tolerance, even in adjusted analyses. WSB = within-study bias; RB = reporting bias; IND = indirectness; IMP = imprecision; HET = heterogeneity; INC = incoherence. k = number of studies providing direct evidence for the comparison. HR = network hazard ratio from the primary adjusted-hazard-ratio network (random-effects model); estimates and confidence ratings therefore correspond to the primary HR-only network. Imprecision was re-evaluated for the HR-only estimates against the pre-specified clinically important risk-ratio thresholds of 0.90 and 1.11; comparisons whose 95% CI crossed unity and spanned both thresholds were downgraded for imprecision. The three quadruple-therapy cardiovascular-mortality comparisons are not estimable in the HR-only network (the corresponding all-measures estimates are reported in Supplemental Table S7). Confidence ratings follow the CINeMA framework: High (no downgrading), Moderate (≤1 domain with some concerns), Low (≥1 domain with major concerns or multiple domains with some concerns), Very low (multiple domains with major concerns or extensive downgrading). QT = quadruple therapy; TT = triple therapy; DT = double therapy; S/N = single/none therapy.

Supplemental Table S6. CINeMA Domain-Specific Judgments and Reasons for Downgrading

Outcome	*Comparison*	*Confidence*	*Reasons for Downgrading*
All-Cause Mortality	QT vs S/N	Very low	Within-study bias, Reporting bias, Indirectness, Imprecision
	TT vs S/N	Low	Within-study bias, Reporting bias, Indirectness
	DT vs S/N	Low	Within-study bias, Reporting bias, Indirectness
	QT vs TT	Very low	Within-study bias, Reporting bias, Indirectness, Imprecision
	QT vs DT	Very low	Within-study bias, Reporting bias, Indirectness, Imprecision

	TT vs DT	Very low	Within-study bias, Reporting bias, Indirectness, Imprecision
Composite ACM + HFH	QT vs S/N	Very low	Within-study bias, Reporting bias, Indirectness
	TT vs S/N	Low	Within-study bias, Reporting bias, Indirectness
	DT vs S/N	Low	Within-study bias, Reporting bias, Indirectness
	QT vs TT	Very low	Within-study bias, Reporting bias, Indirectness, Imprecision
	QT vs DT	Low	Within-study bias, Reporting bias, Indirectness, Imprecision
	TT vs DT	Low	Within-study bias, Reporting bias, Indirectness, Imprecision
HF Hospitalization	QT vs S/N	Low	Within-study bias, Reporting bias, Indirectness
	TT vs S/N	Very low	Within-study bias, Reporting bias, Indirectness, Heterogeneity
	DT vs S/N	Very low	Within-study bias, Reporting bias, Indirectness, Heterogeneity
	QT vs TT	Very low	Within-study bias, Reporting bias, Indirectness, Imprecision, Heterogeneity
	QT vs DT	Very low	Within-study bias, Reporting bias, Indirectness, Imprecision, Heterogeneity
	TT vs DT	Very low	Within-study bias, Reporting bias, Indirectness, Imprecision
CV Mortality	QT vs S/N	Not estimable	Not estimable in the HR-only network (quadruple node not connected to this comparator)
	TT vs S/N	Low	Within-study bias, Reporting bias, Indirectness, Imprecision
	DT vs S/N	Very low	Within-study bias, Reporting bias, Indirectness, Imprecision, Heterogeneity
	QT vs TT	Not estimable	Not estimable in the HR-only network (quadruple node not connected to this comparator)

	QT vs DT	Not estimable	Not estimable in the HR-only network (quadruple node not connected to this comparator)
	TT vs DT	Very low	Within-study bias, Reporting bias, Indirectness, Imprecision, Heterogeneity

Confidence was assessed using the CINeMA (Confidence in Network Meta-Analysis) framework across six domains. Within-study bias was rated as major concerns for all comparisons due to the predominantly observational evidence base (26 of 27 studies). Reporting bias was rated as some concerns throughout due to the absence of a comprehensive grey-literature search and the possibility of unpublished negative studies. Indirectness was rated as some concerns for most comparisons because of population heterogeneity (inclusion of HFmrEF in some studies) and variability in GDMT treatment constructs across studies. Imprecision was judged against pre-specified clinically important thresholds (RR 0.90–1.11). Heterogeneity was assessed by comparing confidence and prediction intervals with the range of equivalence. Incoherence was assessed using node-splitting (SIDE approach) and design-based decomposition of Q-statistics.

Supplemental Table S7. Sensitivity Analyses: Time-Stratified and HR-Only Results vs Single/None

Outcome	Time Window	Measures	Comparison	Effect (95% CI)	k	n	Rows	τ^2	p-het
**Composite ACM + HFH**	30–90 days	HR only	Triple vs Single/None	0.680 [0.515; 0.898]	1	3	3	—	—
			Double vs Single/None	0.800 [0.632; 1.013]	1	3	3	—	—
			Quadruple vs Single/None	0.520 [0.360; 0.751]	1	3	3	—	—
		All measures	Triple vs Single/None	0.680 [0.515; 0.898]	1	3	3	—	—
			Double vs Single/None	0.800 [0.632; 1.013]	1	3	3	—	—
			Quadruple vs Single/None	0.520 [0.360; 0.751]	1	3	3	—	—
	6–12 months	HR only	Triple vs Single/None	0.750 [0.602; 0.934]	1	2	2	—	—
			Double vs Single/None	0.773 [0.573; 1.044]	1	2	2	—	—
		All measures	Triple vs Single/None	0.667 [0.588; 0.756]	3	6	6	0.0000	0.493

			Double vs Single/None	0.709 [0.613; 0.820]	3	6	6	0.0000	0.493
	>12 months	HR only	Triple vs Single/None	0.700 [0.544; 0.900]	2	3	3	0.0000	0.498
			Double vs Single/None	0.749 [0.660; 0.849]	2	3	3	0.0000	0.498
		All measures	Triple vs Single/None	0.700 [0.544; 0.900]	2	3	3	0.0000	0.498
			Double vs Single/None	0.749 [0.660; 0.849]	2	3	3	0.0000	0.498
	Overall	HR only	Triple vs Single/None	0.721 [0.639; 0.814]	4	8	8	0.0000	0.956
			Double vs Single/None	0.756 [0.683; 0.837]	4	8	8	0.0000	0.956
			Quadruple vs Single/None	0.520 [0.360; 0.751]	4	8	8	0.0000	0.956
**HF hospitalization**	30–90 days	HR only	Triple vs Single/None	0.780 [0.555; 1.096]	3	8	8	0.0293	0.167
			Double vs Single/None	0.768 [0.576; 1.025]	3	8	8	0.0293	0.167
			Quadruple vs Single/None	0.635 [0.432; 0.936]	3	8	8	0.0293	0.167
		All measures	Triple vs Single/None	0.780 [0.555; 1.096]	3	8	8	0.0293	0.167
			Double vs Single/None	0.768 [0.576; 1.025]	3	8	8	0.0293	0.167
			Quadruple vs Single/None	0.635 [0.432; 0.936]	3	8	8	0.0293	0.167
	6–12 months	HR only	Triple vs Single/None	0.869 [0.705; 1.072]	3	5	5	0.0000	0.877
			Double vs Single/None	1.007 [0.786; 1.290]	3	5	5	0.0000	0.877

		All measures	Triple vs Single/None	0.761 [0.540; 1.072]	4	6	11	0.0537	0.089
			Double vs Single/None	0.986 [0.684; 1.422]	4	6	11	0.0537	0.089
			Quadruple vs Single/None	0.354 [0.159; 0.785]	4	6	11	0.0537	0.089
	>12 months	HR only	Triple vs Single/None	0.650 [0.488; 0.866]	3	4	4	0.0000	0.642
			Double vs Single/None	0.731 [0.638; 0.839]	3	4	4	0.0000	0.642
		All measures	Triple vs Single/None	0.650 [0.488; 0.866]	3	4	4	0.0000	0.642
			Double vs Single/None	0.731 [0.638; 0.839]	3	4	4	0.0000	0.642
	Overall	HR only	Triple vs Single/None	0.762 [0.664; 0.875]	9	17	17	0.0041	0.314
			Double vs Single/None	0.789 [0.705; 0.883]	9	17	17	0.0041	0.314
			Quadruple vs Single/None	0.638 [0.468; 0.869]	9	17	17	0.0041	0.314
**CV mortality**	6–12 months	HR only	Triple vs Single/None	0.665 [0.487; 0.906]	2	3	3	0.0000	0.634
			Double vs Single/None	0.624 [0.455; 0.858]	2	3	3	0.0000	0.634
		All measures	Triple vs Single/None	0.537 [0.372; 0.776]	3	5	5	0.0402	0.159
			Double vs Single/None	0.562 [0.388; 0.815]	3	5	5	0.0402	0.159
	>12 months	HR only	Triple vs Single/None	0.383 [0.125; 1.174]	2	3	3	0.0635	0.260
			Double vs Single/None	0.850 [0.371; 1.949]	2	3	3	0.0635	0.260

		All measures	Triple vs Single/None	0.292 [0.144; 0.591]	3	4	9	0.0000	0.535
			Double vs Single/None	0.700 [0.412; 1.189]	3	4	9	0.0000	0.535
			Quadruple vs Single/None	0.073 [0.015; 0.349]	3	4	9	0.0000	0.535
	Overall	HR only	Triple vs Single/None	0.600 [0.381; 0.944]	4	6	6	0.0589	0.138
			Double vs Single/None	0.746 [0.492; 1.130]	4	6	6	0.0589	0.138
**All-cause mortality**	6–12 months	HR only	Triple vs Single/None	0.600 [0.492; 0.730]	8	11	11	0.0006	0.418
			Double vs Single/None	0.550 [0.506; 0.599]	8	11	11	0.0006	0.418
		All measures	Triple vs Single/None	0.495 [0.403; 0.608]	10	14	19	0.0150	0.113
			Double vs Single/None	0.539 [0.470; 0.617]	10	14	19	0.0150	0.113
			Quadruple vs Single/None	0.081 [0.033; 0.198]	10	14	19	0.0150	0.113
	>12 months	HR only	Triple vs Single/None	0.565 [0.435; 0.734]	5	9	9	0.0000	0.777
			Double vs Single/None	0.696 [0.609; 0.795]	5	9	9	0.0000	0.777
			Quadruple vs Single/None	0.400 [0.092; 1.738]	5	9	9	0.0000	0.777
		All measures	Triple vs Single/None	0.565 [0.435; 0.734]	5	9	9	0.0000	0.777
			Double vs Single/None	0.696 [0.609; 0.795]	5	9	9	0.0000	0.777
			Quadruple vs Single/None	0.400 [0.092; 1.738]	5	9	9	0.0000	0.777

	Overall	HR only	Triple vs Single/None	0.585 [0.492; 0.696]	13	20	20	0.0070	0.194
			Double vs Single/None	0.594 [0.539; 0.655]	13	20	20	0.0070	0.194
			Quadruple vs Single/None	0.400 [0.091; 1.754]	13	20	20	0.0070	0.194

Supplemental Table S8. Leave-One-Out Sensitivity Analysis for All-Cause Mortality

Omitted Study	Remaining k	τ^2	Triple vs Single/None	Quadruple vs Single/None
Abe_2020	14	0.0154	0.537 [0.451; 0.640]	0.130 [0.057; 0.295]
Abe_2023	14	0.0180	0.520 [0.437; 0.618]	0.130 [0.057; 0.296]
Ahn_2019	14	0.0198	0.526 [0.437; 0.632]	0.130 [0.057; 0.297]
Busson_2018	14	0.0182	0.520 [0.437; 0.618]	0.130 [0.057; 0.296]
Chen_2021	14	0.0173	0.521 [0.439; 0.619]	0.130 [0.057; 0.296]
Gilstrap_2023	14	0.0234	0.516 [0.430; 0.619]	0.131 [0.057; 0.300]
Grewal_2021	14	0.0203	0.515 [0.431; 0.616]	0.131 [0.057; 0.298]
Huang_2025	14	0.0101	0.546 [0.463; 0.645]	0.400 [0.091; 1.761]
Kawakubo_2022	14	0.0175	0.494 [0.410; 0.596]	0.130 [0.057; 0.296]
Miyoshi_2026	14	0.0116	0.453 [0.369; 0.556]	0.130 [0.058; 0.292]
Oren_2024	14	0.0153	0.517 [0.436; 0.614]	0.080 [0.030; 0.213]
Takeuchi_2022	14	0.0154	0.517 [0.436; 0.612]	0.130 [0.057; 0.295]
Vicent_2019	14	0.0155	0.549 [0.457; 0.658]	0.130 [0.057; 0.295]
Vorilhon_2015	14	0.0210	0.511 [0.424; 0.615]	0.131 [0.057; 0.298]
Yamaguchi_2018	14	0.0112	0.526 [0.447; 0.619]	0.130 [0.058; 0.292]

Leave-one-out estimates from the all-measures (hazard-ratio + risk-ratio) random-effects all-cause-mortality network — the network in which the quadruple node is informed in part by crude risk ratios (Huang 2025) — which comprises 15 studies; the primary HR-only ACM network comprises 13. Values are network hazard/relative-effect ratios [95% CI]. k = number of remaining studies; τ^2 = between-study variance.

Supplemental Table S9. Distribution of Effect Modifiers Across Treatment-Intensity Nodes (Transitivity Assessment)

Characteristic	Double (k=6)	Triple (k=14)	Quadruple (k=7)
Mean age, y	74.6 (71–79)	69.2 (63–84)	65.7 (59–70)
Female, %	34.3 (27–41)	35.2 (21–63)	36.3 (24–76)
Mean LVEF, %	31.0 (28–34)	30.1 (23–36)	28.1 (24–32)
Follow-up, months	14.7 (12–24)	17.6 (6–43)	10.1 (1–36)
Diabetes, %	38.0 (36–40)	36.2 (28–47)	39.3 (16–82)
CKD, %	44.2 (21–68)	25.2 (9–56)	40.5 (36–45)
Mean eGFR, mL/min/1.73m²	51.6 (48–58)	52.4 (41–62)	63.1 (57–68)
Admission SBP, mmHg	118.9 (108–135)	125.6 (112–146)	115.9 (109–122)
Publication era	2017–2023 (≥2021: 50%)	2015–2026 (≥2021: 57%)	2024–2026 (≥2021: 100%)
Geographic region	Asia 4; Europe 1; North America 1	Asia 9; Europe 3; North America 1; Multinational 1	North America 3; Asia 2; South America 1; Europe 1

Values are mean (range) across studies grouped by the most intensive regimen evaluated (index node), except publication era and region (counts). CKD = chronic kidney disease; eGFR = estimated glomerular filtration rate; LVEF = left ventricular ejection fraction; SBP = systolic blood pressure.

Supplemental Table S10. Illustrative Absolute Risk Reduction and NNT Across Baseline-Risk Scenarios (Composite of All-Cause Mortality and HF Hospitalization, HR-only)

Baseline 1-yr risk	Comparison	Treated risk, %	ARR, % (95% CI)	NNT (95% CI)
20%	Double vs Single/None	15.5	4.5 (3.0 to 5.9)	22 (17 to 34)
20%	Triple vs Single/None	14.9	5.1 (3.4 to 6.7)	19 (15 to 30)
25%	Double vs Single/None	19.5	5.5 (3.6 to 7.2)	18 (14 to 28)
25%	Triple vs Single/None	18.7	6.3 (4.1 to 8.2)	16 (12 to 24)
30%	Double vs Single/None	23.6	6.4 (4.2 to 8.4)	16 (12 to 24)
30%	Triple vs Single/None	22.7	7.3 (4.8 to 9.6)	14 (10 to 21)
35%	Double vs Single/None	27.8	7.2 (4.7 to 9.5)	14 (11 to 21)
35%	Triple vs Single/None	26.7	8.3 (5.4 to 10.9)	12 (9 to 18)
40%	Double vs Single/None	32.0	8.0 (5.2 to 10.5)	13 (9 to 19)
40%	Triple vs Single/None	30.8	9.2 (6.0 to 12.2)	11 (8 to 17)
45%	Double vs Single/None	36.4	8.6 (5.6 to 11.5)	12 (9 to 18)
45%	Triple vs Single/None	35.0	10.0 (6.5 to 13.3)	10 (8 to 15)
50%	Double vs Single/None	40.8	9.2 (6.0 to 12.3)	11 (8 to 17)
50%	Triple vs Single/None	39.3	10.7 (6.9 to 14.2)	9 (7 to 15)

Illustrative translations of the primary HR-only composite (all-cause mortality and HF hospitalization) network estimates across assumed baseline 1-year composite risks, computed as treated risk = 1 − (1 − baseline)^HR; they assume the association is causal and constant across baseline risk and are not estimates of achievable benefit. Absolute estimates are presented for double and triple therapy only; an absolute translation is not reported for quadruple therapy because the quadruple-versus-single/none composite estimate is informed by a single direct study (k = 1), which we judged insufficient for a stable absolute-risk projection. ARR = absolute risk reduction; HR = hazard ratio; NNT = number needed to treat.

Supplemental Table S11. Design-Based Decomposition of Heterogeneity (Q-Statistics)

Analysis	Component	Q	df	p-value
**All-cause mortality ∣ 6–12 months ∣ All measures**	Total	19.31	13	0.114
	Within designs	13.88	10	0.179
	Between designs	5.44	3	0.142
**All-cause mortality ∣ 6–12 months ∣ HR only**	Total	9.22	9	0.418
	Within designs	8.98	8	0.344
	Between designs	0.24	1	0.628
**All-cause mortality ∣ >12 months ∣ All measures**	Total	3.25	6	0.777
	Within designs	0.75	5	0.980
	Between designs	2.49	1	0.114
**All-cause mortality ∣ >12 months ∣ HR only**	Total	3.25	6	0.777
	Within designs	0.75	5	0.980
	Between designs	2.49	1	0.114
**All-cause mortality ∣ Overall ∣ All measures**	Total	34.57	22	0.043
	Within designs	26.18	18	0.096
	Between designs	8.39	4	0.078
**All-cause mortality ∣ Overall ∣ HR only**	Total	21.75	17	0.194
	Within designs	20.68	16	0.191
	Between designs	1.07	1	0.301
**Composite ACM + HFH ∣ 6–12 months ∣ All measures**	Total	3.40	4	0.493
	Within designs	3.21	3	0.360
	Between designs	0.19	1	0.662
**Composite ACM + HFH ∣ 6–12 months ∣ HR only**	Total	0.00	0	—
	Within designs	0.00	0	—
	Between designs	0.00	0	—
**Composite ACM + HFH ∣ >12 months ∣ All measures**	Total	0.46	1	0.498
	Within designs	0.46	1	0.498
	Between designs	0.00	0	—
**Composite ACM + HFH ∣ >12 months ∣ HR only**	Total	0.46	1	0.498
	Within designs	0.46	1	0.498
	Between designs	0.00	0	—
**Composite ACM + HFH ∣ Overall ∣ All measures**	Total	4.70	9	0.859
	Within designs	4.28	8	0.831

	Between designs	0.42	1	0.515
**Composite ACM + HFH ǀ Overall ǀ HR only**	Total	1.08	5	0.956
	Within designs	1.03	4	0.905
	Between designs	0.05	1	0.829
**Composite ACM + HFH ǀ 30–90 days ǀ All measures**	Total	0.00	0	—
	Within designs	0.00	0	—
	Between designs	0.00	0	—
**Composite ACM + HFH ǀ 30–90 days ǀ HR only**	Total	0.00	0	—
	Within designs	0.00	0	—
	Between designs	0.00	0	—
**CV mortality ǀ 6–12 months ǀ All measures**	Total	5.17	3	0.159
	Within designs	5.13	2	0.077
	Between designs	0.04	1	0.840
**CV mortality ǀ 6–12 months ǀ HR only**	Total	0.23	1	0.634
	Within designs	0.00	0	—
	Between designs	0.23	1	0.634
**CV mortality ǀ >12 months ǀ All measures**	Total	2.19	3	0.534
	Within designs	1.27	1	0.260
	Between designs	0.92	2	0.631
**CV mortality ǀ >12 months ǀ HR only**	Total	1.27	1	0.260
	Within designs	1.27	1	0.260
	Between designs	0.00	0	—
**CV mortality ǀ Overall ǀ All measures**	Total	14.49	8	0.070
	Within designs	11.51	5	0.042
	Between designs	2.98	3	0.394
**CV mortality ǀ Overall ǀ HR only**	Total	6.96	4	0.138
	Within designs	6.25	3	0.100
	Between designs	0.71	1	0.399
**HF hospitalization ǀ 6–12 months ǀ All measures**	Total	9.54	5	0.089
	Within designs	0.23	2	0.890
	Between designs	9.31	3	0.025
**HF hospitalization ǀ 6–12 months ǀ HR only**	Total	0.68	3	0.877
	Within designs	0.23	2	0.890
	Between designs	0.45	1	0.502
**HF hospitalization ǀ >12 months ǀ All measures**	Total	0.89	2	0.642

	Within designs	0.89	2	0.642
	Between designs	0.00	0	—
**HF hospitalization ǀ >12 months ǀ HR only**	Total	0.89	2	0.642
	Within designs	0.89	2	0.642
	Between designs	0.00	0	—
**HF hospitalization ǀ Overall ǀ All measures**	Total	27.65	17	0.049
	Within designs	15.47	13	0.279
	Between designs	12.19	4	0.016
**HF hospitalization ǀ Overall ǀ HR only**	Total	15.99	14	0.314
	Within designs	15.47	13	0.279
	Between designs	0.52	1	0.469
**HF hospitalization ǀ 30–90 days ǀ All measures**	Total	7.81	5	0.167
	Within designs	7.81	5	0.167
	Between designs	0.00	0	—
**HF hospitalization ǀ 30–90 days ǀ HR only**	Total	7.81	5	0.167
	Within designs	7.81	5	0.167
	Between designs	0.00	0	—

Q = Cochran Q statistic; df = degrees of freedom. ALL = all effect measures; HR ONLY = adjusted hazard ratios only. A significant p-value for the between-designs component indicates inconsistency between direct and indirect evidence.

Supplemental Table S12. Alternative Network: Sensitivity Analyses vs Incomplete Therapy

Outcome	Time Window	Measures	Comparison	Effect (95% CI)	k	n	Rows	τ^2	p-het
**Composite ACM + HFH**	6–12 months	HR only	Triple vs Incomplete	0.613 [0.459; 0.818]	5	5	5	0.0000	0.611
			Double vs Incomplete	0.632 [0.444; 0.901]	5	5	5	0.0000	0.611
			Quadruple vs Incomplete	0.499 [0.341; 0.731]	5	5	5	0.0000	0.611
		All measures	Triple vs Incomplete	0.638 [0.523; 0.777]	6	6	6	0.0000	0.772
			Double vs Incomplete	0.657 [0.494; 0.874]	6	6	6	0.0000	0.772

			Quadruple vs Incomplete	0.499 [0.341; 0.731]	6	6	6	0.0000	0.772
	Overall	HR only	Triple vs Incomplete	0.613 [0.459; 0.818]	5	5	5	0.0000	0.611
			Double vs Incomplete	0.632 [0.444; 0.901]	5	5	5	0.0000	0.611
			Quadruple vs Incomplete	0.499 [0.341; 0.731]	5	5	5	0.0000	0.611
**HF hospitalization**	6–12 months	HR only	Triple vs Incomplete	0.650 [0.435; 0.971]	3	3	3	—	—
			Double vs Incomplete	0.722 [0.451; 1.156]	3	3	3	—	—
			Quadruple vs Incomplete	0.260 [0.111; 0.607]	3	3	3	—	—
		All measures	Triple vs Incomplete	0.578 [0.383; 0.874]	5	5	7	0.0586	0.145
			Double vs Incomplete	0.780 [0.429; 1.417]	5	5	7	0.0586	0.145
			Quadruple vs Incomplete	0.319 [0.159; 0.640]	5	5	7	0.0586	0.145
	Overall	HR only	Triple vs Incomplete	0.650 [0.435; 0.971]	3	3	3	—	—
			Double vs Incomplete	0.722 [0.451; 1.156]	3	3	3	—	—
			Quadruple vs Incomplete	0.260 [0.111; 0.607]	3	3	3	—	—
**CV mortality**	6–12 months	All measures	Triple vs Incomplete	0.740 [0.471; 1.163]	3	3	3	—	—
			Double vs Incomplete	0.733 [0.406; 1.321]	3	3	3	—	—
			Quadruple vs Incomplete	1.220 [0.507; 2.934]	3	3	3	—	—

**All-cause mortality**	6–12 months	HR only	Triple vs Incomplete	0.590 [0.358; 0.973]	2	2	2	—	—
			Double vs Incomplete	0.573 [0.320; 1.026]	2	2	2	—	—
		All measures	Triple vs Incomplete	0.712 [0.397; 1.275]	4	4	6	0.1236	0.048
			Double vs Incomplete	0.945 [0.412; 2.168]	4	4	6	0.1236	0.048
			Quadruple vs Incomplete	0.140 [0.039; 0.494]	4	4	6	0.1236	0.048
	>12 months	HR only	Triple vs Incomplete	0.654 [0.500; 0.856]	3	3	3	0.0000	0.507
			Double vs Incomplete	1.235 [0.641; 2.379]	3	3	3	0.0000	0.507
		All measures	Triple vs Incomplete	0.654 [0.500; 0.856]	3	3	3	0.0000	0.507
			Double vs Incomplete	1.235 [0.641; 2.379]	3	3	3	0.0000	0.507
	Overall	HR only	Triple vs Incomplete	0.629 [0.464; 0.851]	5	5	5	0.0240	0.225
			Double vs Incomplete	0.739 [0.461; 1.182]	5	5	5	0.0240	0.225

Abbreviations as in Supplemental Table S4. Network estimates from the alternative four-node model using incomplete therapy (less comprehensive therapy than the treatment being evaluated, but could not be mapped confidently to a fixed number of GDMT pillars) as the reference. This analysis provides a complementary perspective to the primary network anchored to single/none therapy.

Supplemental Table S13. Proportion of Direct versus Indirect Evidence for Major Comparisons (Primary HR-only Networks)

Outcome	Comparison	Network HR	Direct evidence, %
All-cause mortality	Double vs Single/None	0.594	93%
All-cause mortality	Quadruple vs Single/None	0.4	100%
All-cause mortality	Quadruple vs Triple	0.683	0%
All-cause mortality	Triple vs Single/None	0.585	70%
Composite ACM + HFH	Double vs Single/None	0.756	83%
Composite ACM + HFH	Quadruple vs Single/None	0.52	100%
Composite ACM + HFH	Quadruple vs Triple	0.721	0%

Composite ACM + HFH	Triple vs Single/None	0.721	73%
HF hospitalization	Double vs Single/None	0.789	87%
HF hospitalization	Quadruple vs Single/None	0.638	100%
HF hospitalization	Quadruple vs Triple	0.837	0%
HF hospitalization	Triple vs Single/None	0.762	79%
Cardiovascular mortality	Double vs Single/None	0.746	71%
Cardiovascular mortality	Triple vs Single/None	0.6	56%

Proportion of information contributed by direct evidence to each network estimate in the primary (adjusted hazard ratio) networks, from node-splitting. Quadruple-versus-triple comparisons derived 0% from direct evidence (entirely indirect), and quadruple-versus-single/none rested on a single direct study. The full percentage contribution matrix for all-cause mortality is shown in Supplemental Figure S4.

SUPPLEMENTAL FIGURES

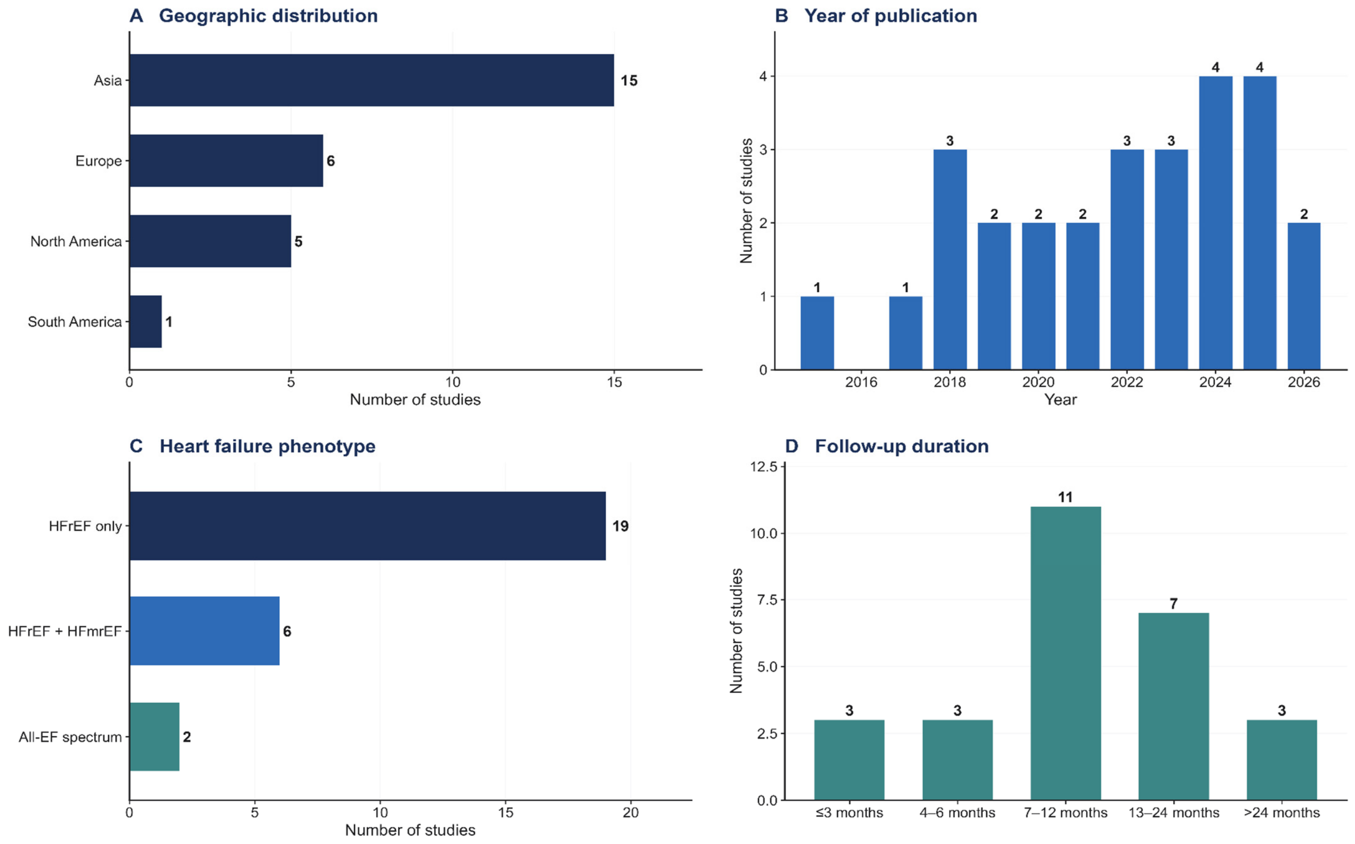

Supplemental Figure S1. Characteristics of the 27 included studies.

Distribution of included studies by (A) geographic region, (B) year of publication, (C) heart failure phenotype, and (D) follow-up duration. Numbers above or beside each bar indicate the count of studies in that category. HFmrEF = heart failure with mildly reduced ejection fraction; HFrEF = heart failure with reduced ejection fraction.

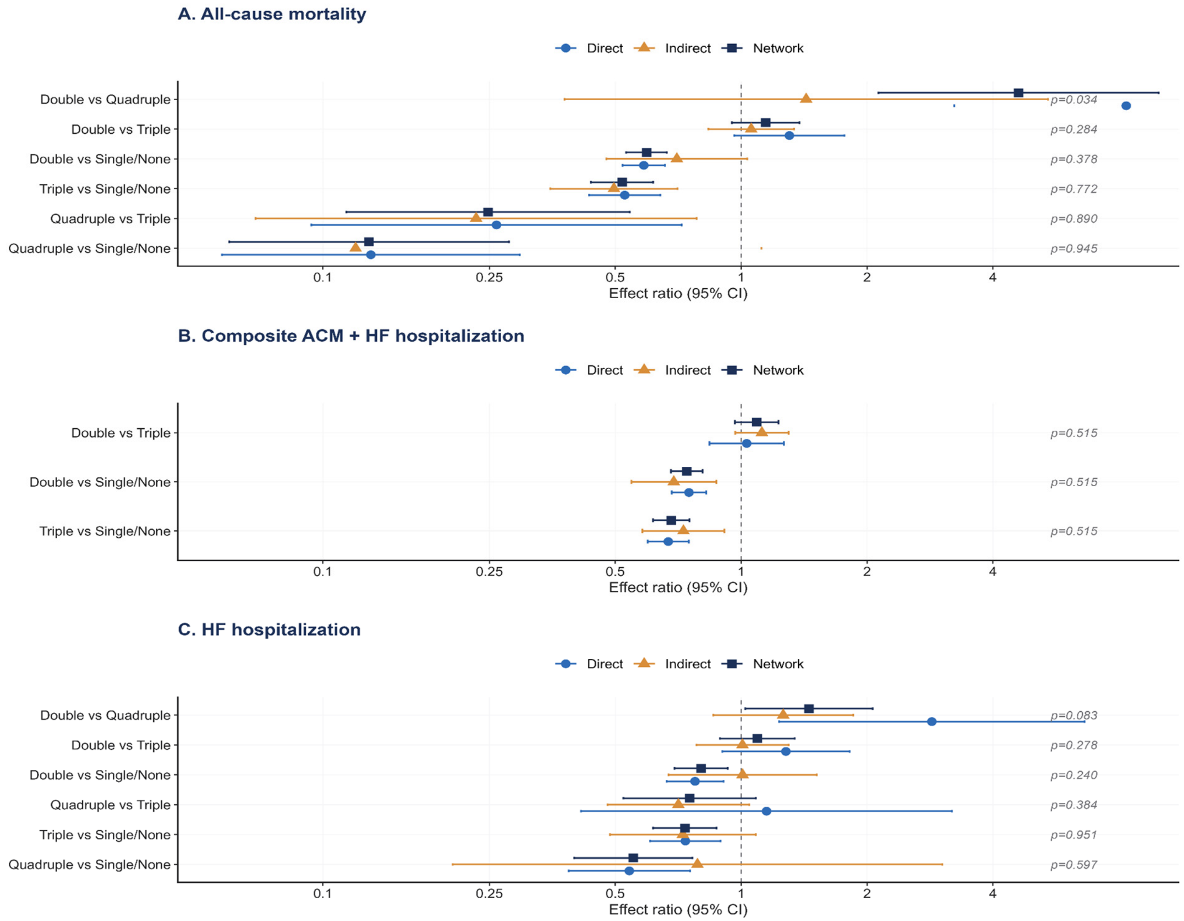

Supplemental Figure S2. Node-splitting assessment of local inconsistency.

Random-effects direct, indirect, and network (combined) relative effect estimates with 95% confidence intervals are shown for each splittable comparison in the primary networks for (A) all-cause mortality, (B) the composite of all-cause mortality and heart failure hospitalization, and (C)

heart failure hospitalization. P-values to the right of each row test the disagreement between direct and indirect evidence using the SIDE (Separating Indirect from Direct Evidence) approach; a p-value <0.05 indicates statistically significant local inconsistency. The cardiovascular mortality network is not shown because it contains no splittable contrasts (no closed loops with both direct and indirect evidence). ACM = all-cause mortality; CI = confidence interval; HFH = heart failure hospitalization.

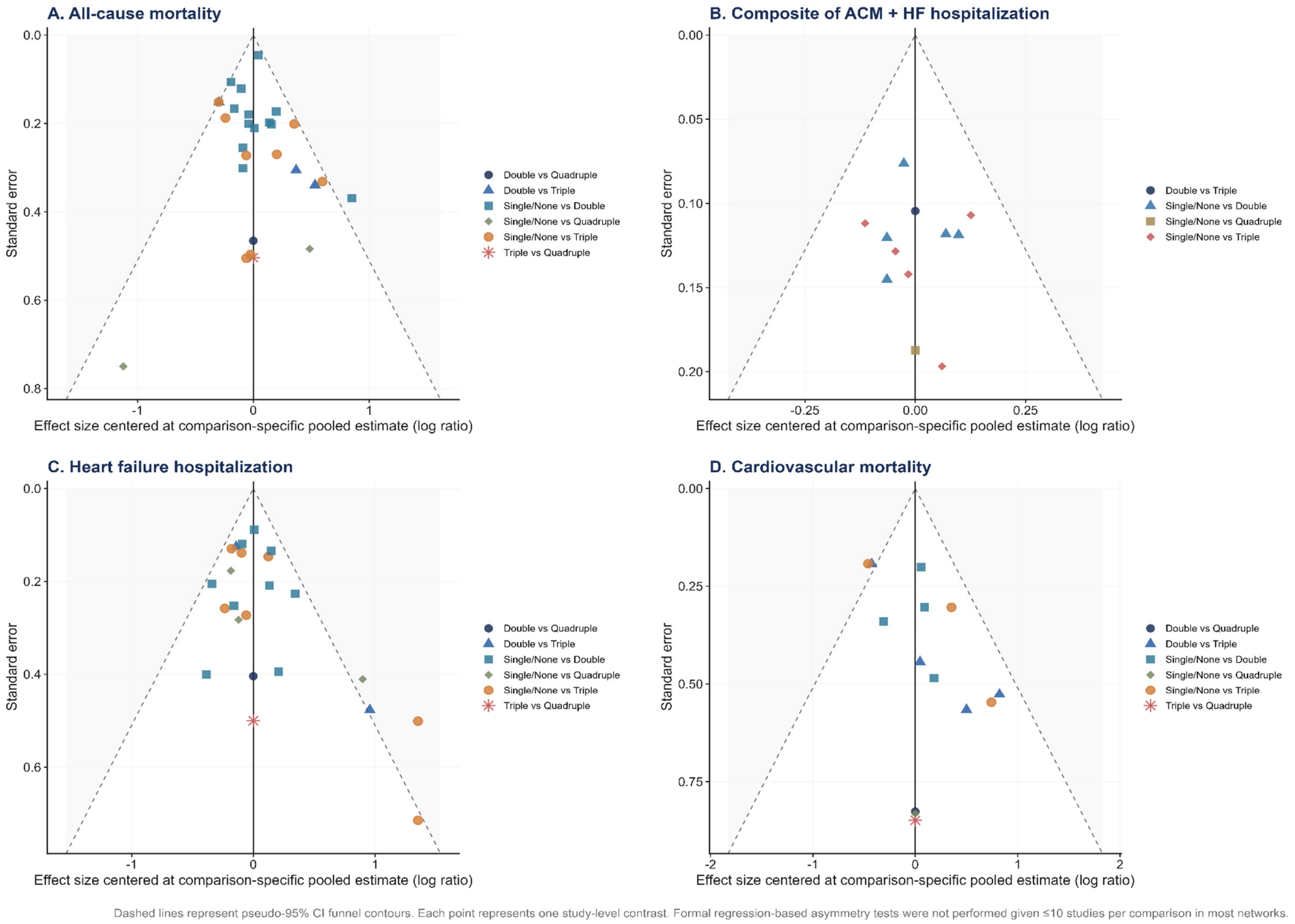

Supplemental Figure S3. Comparison-adjusted funnel plots for all primary outcomes.

Each panel shows study-level contrasts for (A) all-cause mortality, (B) the composite of all-cause mortality and heart failure hospitalization, (C) heart failure hospitalization, and (D) cardiovascular mortality. Effect sizes are centered at the comparison-specific pooled estimate, so the vertical reference

line (x = 0) corresponds to no deviation from the pooled estimate. Treatments are ordered by intensity (single/none → double → triple → quadruple) such that asymmetry to the left of the reference line would indicate small-study effects favoring more intensive therapy. Dashed lines represent pseudo-95% confidence interval funnel contours. Each point represents one study-level contrast, color-coded by the comparison type. Formal regression-based asymmetry tests were not performed given the small number of studies per comparison. CI = confidence interval; DT = double therapy; QT = quadruple therapy; ST/NT = single/no therapy; TT = triple therapy.

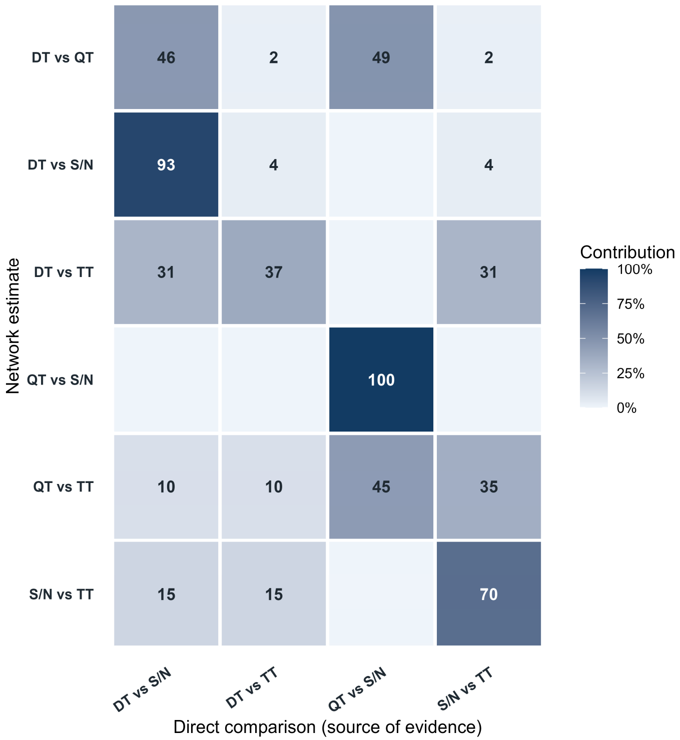

Supplemental Figure S4. Percentage Contribution Matrix (Primary HR-only All-Cause Mortality Network).

Each row shows the percentage of information for a network estimate contributed by each direct comparison (column). Quadruple-versus-single/none is informed entirely by its single direct study, whereas quadruple-versus-triple and quadruple-versus-double draw on indirect paths only.

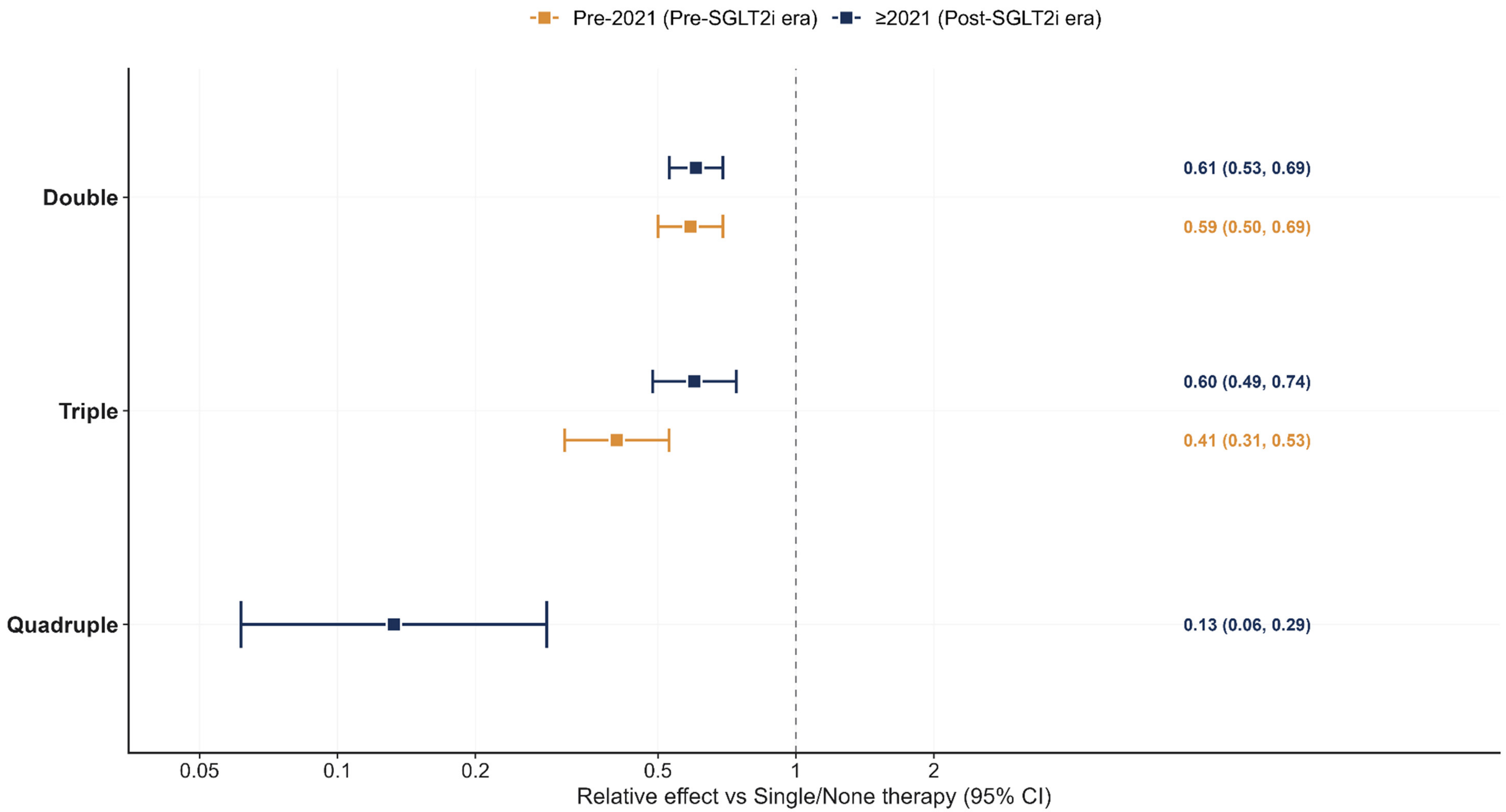

Supplemental Figure S5. Era-stratified all-cause mortality network meta-analysis.

Random-effects network estimates versus single/no therapy from the primary 4-node network, stratified by publication era (pre-2021 vs ≥2021). The 2021 cutoff was chosen because it marked the widespread guideline adoption of SGLT2 inhibitors as a fourth pillar of HFrEF therapy. Quadruple-

therapy evidence is absent from the pre-2021 era because no eligible studies from that period reported outcomes for this treatment node, reflecting the clinical adoption timeline of SGLT2 inhibitors. Effects are relative effects (log-ratio scale) presented with 95% CIs. CI = confidence interval.
